# The return of metabolism: biochemistry and physiology of the pentose phosphate pathway

**DOI:** 10.1111/brv.12140

**Published:** 2014-09-22

**Authors:** Anna Stincone, Alessandro Prigione, Thorsten Cramer, Mirjam M. C. Wamelink, Kate Campbell, Eric Cheung, Viridiana Olin-Sandoval, Nana-Maria Grüning, Antje Krüger, Mohammad Tauqeer Alam, Markus A. Keller, Michael Breitenbach, Kevin M. Brindle, Joshua D. Rabinowitz, Markus Ralser

**Affiliations:** 1 Department of Biochemistry, University of Cambridge, 80 Tennis Court Road, Cambridge CB2 1GA, U.K; 2 Cambridge Systems Biology Centre, University of Cambridge, 80 Tennis Court Road, Cambridge CB2 1GA, U.K; 3 Max Delbrueck Centre for Molecular Medicine, Robert-Rössle-Str. 10, 13092 Berlin, Germany; 4 Department of Gastroenterology and Hepatology, Molekulares Krebsforschungszentrum (MKFZ), Charité - Universitätsmedizin Berlin, Campus Virchow-Klinikum, Augustenburger Platz 1, 13353 Berlin, Germany; 5 Metabolic Unit, Department of Clinical Chemistry, VU University Medical Centre Amsterdam, De Boelelaaan 1117, 1081 HV, Amsterdam, The Netherlands; 6 Cancer Research UK, Beatson Institute, Switchback Road, Glasgow G61 1BD, U.K; 7 Max Planck Institute for Molecular Genetics, Ihnestr 73, 14195 Berlin, Germany; 8 Department of Cell Biology, University of Salzburg, Hellbrunnerstrasse 34, A-5020, Salzburg, Austria; 9 Cancer Research UK Cambridge Research Institute (CRI), Li Ka Shing Centre, University of Cambridge, Robinson Way, Cambridge CB2 0RE, U.K; 10 Department of Chemistry, Lewis-Sigler Institute for Integrative Genomics, Princeton University, Princeton, 08544 NJ, U.S.A; 11 Division of Physiology and Metabolism, MRC National Institute for Medical Research, The Ridgeway, Mill Hill, London, NW7, U.K

**Keywords:** pentose phosphate pathway, glycolysis, glucose 6-phosphate dehydrogenase, NADPH, metabolomics, oxidative stress, cancer, stem cells, host–pathogen interactions, metabolic engineering, inherited metabolic disease, parasitic protozoa, metabolism of infection

## Abstract

The pentose phosphate pathway (PPP) is a fundamental component of cellular metabolism. The PPP is important to maintain carbon homoeostasis, to provide precursors for nucleotide and amino acid biosynthesis, to provide reducing molecules for anabolism, and to defeat oxidative stress. The PPP shares reactions with the Entner–Doudoroff pathway and Calvin cycle and divides into an oxidative and non-oxidative branch. The oxidative branch is highly active in most eukaryotes and converts glucose 6-phosphate into carbon dioxide, ribulose 5-phosphate and NADPH. The latter function is critical to maintain redox balance under stress situations, when cells proliferate rapidly, in ageing, and for the ‘Warburg effect’ of cancer cells. The non-oxidative branch instead is virtually ubiquitous, and metabolizes the glycolytic intermediates fructose 6-phosphate and glyceraldehyde 3-phosphate as well as sedoheptulose sugars, yielding ribose 5-phosphate for the synthesis of nucleic acids and sugar phosphate precursors for the synthesis of amino acids. Whereas the oxidative PPP is considered unidirectional, the non-oxidative branch can supply glycolysis with intermediates derived from ribose 5-phosphate and *vice versa*, depending on the biochemical demand. These functions require dynamic regulation of the PPP pathway that is achieved through hierarchical interactions between transcriptome, proteome and metabolome. Consequently, the biochemistry and regulation of this pathway, while still unresolved in many cases, are archetypal for the dynamics of the metabolic network of the cell. In this comprehensive article we review seminal work that led to the discovery and description of the pathway that date back now for 80 years, and address recent results about genetic and metabolic mechanisms that regulate its activity. These biochemical principles are discussed in the context of PPP deficiencies causing metabolic disease and the role of this pathway in biotechnology, bacterial and parasite infections, neurons, stem cell potency and cancer metabolism.

## I. INTRODUCTION

Next to glycolysis (Embden–Meyerhof–Parnas pathway) and the tricarboxylic acid (Krebs) cycle, the pentose phosphate pathway (PPP) was one of the first metabolic pathways to be discovered. Work on the PPP was stimulated by the famous Otto Warburg laboratory in Berlin-Dahlem. In the 1930s Warburg demonstrated that the pyridine nucleotide diphosphopyridine nucleotide DPN (now known as NAD^+^) functions as an electron carrier (Warburg, Christian & Griese, 1935; Warburg & Christian, 1936). In addition, this work revealed the existence of a second coenzyme, termed triphosphopyridine nucleotide TPN (now widely known as NADP^+^), that is required for the oxidation of glucose 6-phosphate to 6-phosphogluconate, by an enzyme which was purified from yeast and erythrocytes and named *Zwischenferment* [‘intermediate enzyme’ now glucose 6-phosphate dehydrogenase (G6PDH)] (Warburg *et al.,* 1935; Warburg & Christian, 1936; Dickens, 1938). The TPN dependence of the Zwischenferment led to the speculation that there might be a pathway parallel to glycolysis, involved in the direct oxidation of glucose (reviewed by (Horecker, 2002)). Work in the subsequent three decades, driven substantially by Bernard Horecker at Cornell University, but with important contributions by other leading biochemists including Arthur Kornberg, Terry Wood, Frank Dickens, Fritz Lipmann, Severo Ochoa, Hans Klenow and others, yielded a draft version of the pathway that was presented in 1955 (Gunsalus, Horecker & Wood, 1955). However, it took further decades to complete the canonical pathway map as we know it today, with some enzymes being added only recently [i.e. sedoheptulokinase (SHPK) in humans (Wamelink *et al.,* 2008*b*) and sedoheptulose 1,7 bisphosphatase (SH17BPase) in yeast (Clasquin *et al.,* 2011)]. Meanwhile, the PPP has gained recognition as being a central player in cellular biosynthetic metabolism and in controlling and maintaining the redox homeostasis of cells. As such, it has been implicated in several human diseases including metabolic syndrome, neurodegeneration (Alzheimer’s disease), cardiovascular disease, parasite infections and cancer (Wood, 1985; Zimmer, 1992; Zimmer, 2001; Schaaff-Gerstenschlager & Zimmermann, 1993; Gupte, 2008; Mayr *et al.,* 2008; Orešič *et al.,* 2011; Vander Heiden *et al.,* 2011; Riganti *et al.,* 2012; Wallace, 2012).

## II. BIOCHEMISTRY AND EVOLUTIONARY ORIGIN OF THE PENTOSE PHOSPHATE PATHWAY

**Fig. 1:**
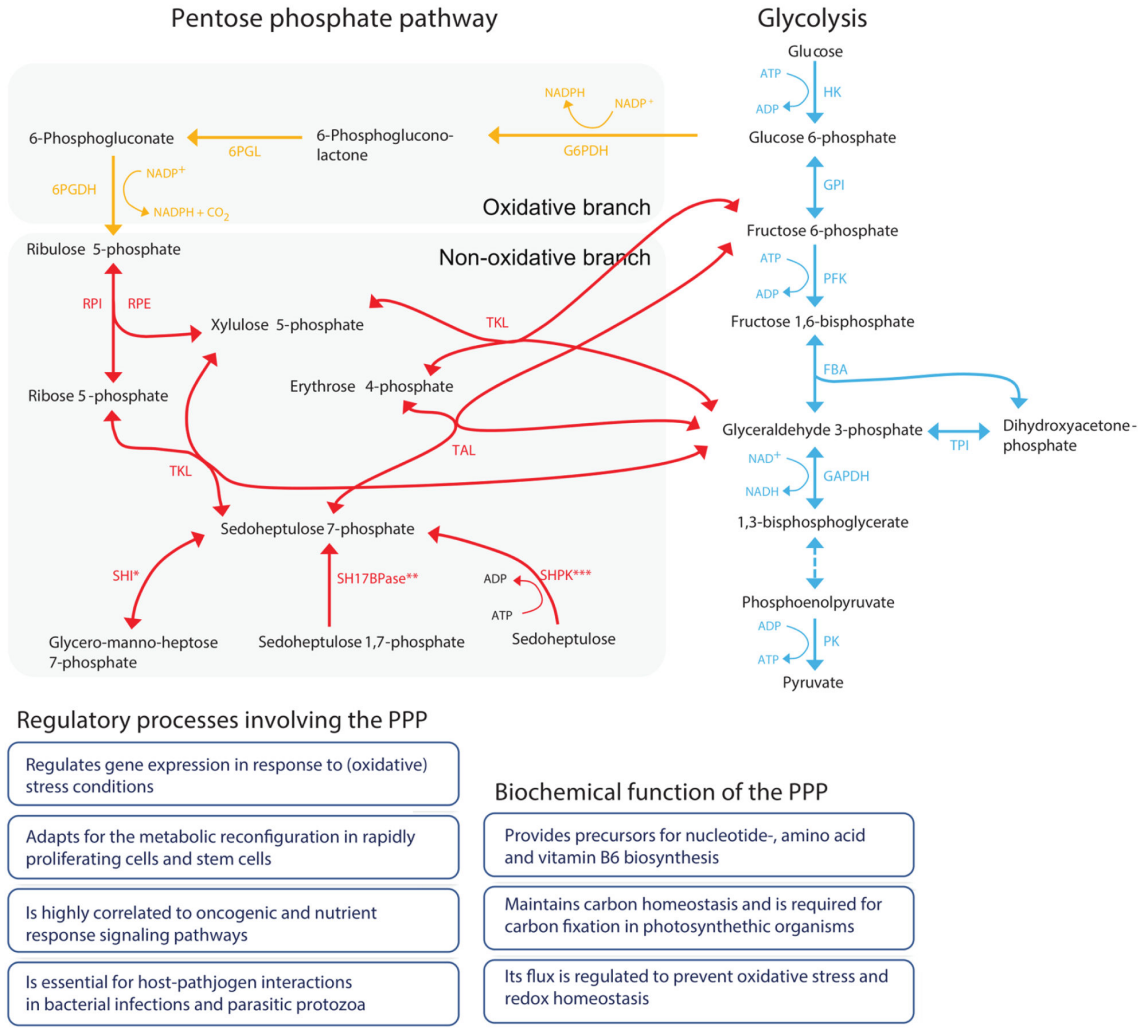
Schematic representation of the pentose phosphate pathway (PPP, left) and glycolysis (canonical topology of the Embden-Meyerhof-Parnas pathway) (right). The enzymatic reactions constituting both pathways are represented by double or single arrows, according to the reversibility of the reactions. The oxidative and non-oxidative branches of the PPP are highlighted by background coloring. Sedoheptulose conversion enzymes found in *bacteria; **fungi (*S. cerevisiae*) and plants, ***mammals. Abbreviations are defined in Table 1; FBA, fructose bisphosphate aldolase; HK, hexokinase; PFK, phosphofructokinase; PK, pyruvate kinase; SH17BP, SH17BPase.

The biochemical reactions that constitute the PPP are, evolutionarily speaking, very old, and seem to accompany life since the earliest steps of evolution. Indeed, metal-catalysed enzyme-free reactions analogous to the PPP are observed in a reconstructed reaction milieu of the prebiotic Archean ocean. This indicates that the basic structure of the PPP is of pre-enzymatic origin and may descend from chemically constraint pre-biotic metal-catalysed sugar phosphate interconversions (Keller, Turchyn & Ralser, 2014). The modern cellular PPP however is catalysed by sophisticated enzymes, except one step, the interconversion of 6-phosphoglucono-*δ*-lactone to 6-phosphogluconate, which is still considered at least partly spontaneous (Wood, 1985; Horecker, 2002). These enzymatic reactions subdivide the PPP into two biochemical branches, known as the oxidative and non-oxidative PPP (see Fig. 1 for an overview of the pathway, and Table 1 for its enzymes).

Reactions of the non-oxidative PPP (with the overlapping Calvin cycle and Entner–Doudoroff pathways), occur virtually ubiquitously, and maintain a central metabolic role in providing the RNA backbone precursors ribose 5-phosphate and erythrose 4-phosphate as precursors for aromatic amino acids. By contrast, the oxidative branch of the PPP is not universal and is absent in many aerobic and thermophilic organisms (Grochowski, Xu & White, 2005; Nunoura *et al.,* 2011; Bräsen *et al.,* 2014). While reactions of the non-oxidative branch can also occur non-enzymatically, reactions concerning the interconversion of glucose 6-phosphate to 6-phosphogluconate, defining the oxidative PPP, were not observed in the Archean ocean simulations (Keller *et al.,* 2014). This observation might indicate that the oxidative part of the PPP pathway is evolutionarily newer than the non-oxidative branch. Nonetheless, in the majority of eukaryotes the oxidative branch is highly active and converts the glycolytic/gluconeogenetic metabolite glucose 6-phosphate into ribulose 5-phosphate *via* the consecutive reactions of G6PDH [in yeast still named Zwf1 (ZWischenFerment) in acknowledgement of Otto Warburg’s original nomenclature], 6-phosphogluconolactonase (6PGL) [catalysing a reaction which can also occur spontaneously but the enzyme increases its specificity (Miclet *et al.,* 2001)] and 6-phosphogluconate dehydrogenase (6PGDH). This metabolic sequence yields two NADPH per metabolized glucose 6-phosphate. Next, the formed ribulose 5-phosphate enters the non-oxidative branch and can be converted either to ribose 5-phosphate by ribose 5-phosphate isomerase (RPI) or to xylulose 5-phosphate by ribulose 5-phosphate epimerase (RPE). While ribose 5-phosphate is required to form the RNA and DNA backbone, erythrose 4-phosphate is required as precursor for the biosynthesis of histidine, for various aromatic metabolites in aromatic amino acid prototrophic organisms and it plays a role in vitamin B6 metabolism (Zimmer, 1992; Wang, Xie & Schultz, 2006; Cadière *et al.,* 2011; Clasquin *et al.,* 2011; Zhao *et al.,* 1995).

The RPI and RPE reactions set the stage for completing the pathway though conversion of ribose 5-phosphate and xylulose 5-phosphate and the glycolytic/gluconeogenetic intermediates glyceraldehyde 3-phosphate and fructose 6-phosphate *via* reshuffling of the monophosphate sugars. These reactions are catalysed by the two enzymes transketolase (TKL) and transaldolase (TAL), which are responsible for relatively complex (multi-substrate) interconversion reactions at the core of the non-oxidative PPP (Fig. 1).

TKL uses a ketose donor (xylulose 5-phosphate) and aldose acceptors (ribose 5-phosphate or erythrose 4-phosphate) to form aldose and ketose products (glyceraldehyde 3-phosphate and sedoheptulose 7-phosphate or fructose 6-phosphate, respectively), to catalyse the transfer of two-carbon fragments (‘activated glycolaldehyde’) for monosaccharide interconversion (Schenk, Duggleby & Nixon, 1998). Hence, this enzyme is responsible for two distinct reactions within the non-oxidative PPP. TKL activity is dependent on the cofactor thiamine diphosphate (Lindqvist *et al.,* 1992; Schenk *et al.,* 1998; Kochetov & Sevostyanova, 2005). The cofactor is bound at the interface between the two subunits of TKL a homodimer, with two identical catalytic sites (Lindqvist *et al.,* 1992; Kochetov & Sevostyanova, 2005).

TAL instead catalyses the transfer of the three-carbon fragment dihydroxyacetone between sugar phosphates up to eight carbons in length *via* the formation of a Schiff base at a lysine residue in the active site (Miosga *et al.,* 1993; Banki & Perl, 1996; Samland & Sprenger, 2009). Its donor substrates are ketose sugar phosphates which include fructose 6-phosphate and sedoheptulose 7-phosphate and its acceptor substrates are the aldose sugar phosphates glyceraldehyde 3-phosphate and erythrose 4-phosphate (Samland & Sprenger, 2009).

By sharing these intermediate metabolites with glycolysis (fructose 6-phosphate and glyceraldehyde 3-phosphate), TAL and TKL act as a bridge between glycolysis and the PPP. In addition, they connect to sedoheptulose 7-phosphate which is synthesized also by other sources. These include the recently described enzyme sedoheptulokinase [SHPK, also known under the former systematic name carbohydrate kinase-like (CARKL)] in mammals (Kardon *et al.,* 2008; Wamelink *et al.,* 2008*b*). SHPK catalyses the phosphorylation of sedoheptulose to sedoheptulose 7-phosphate though ATP consumption in a biochemical reaction first described in 1955 (Ebata, Sato & Bak, 1955). Other novel enzymes that metabolize sedoheptulose 7-phosphate are sedoheptulose 1,7-bisphosphatase (SH17BPase) in yeast (Clasquin *et al.,* 2011) and sedoheptulose 7-phosphate isomerase (SHI) in bacteria (Kneidinger *et al.,* 2001; Valvano, Messner & Kosma, 2002; Taylor *et al.,* 2008). Hence, sedoheptulose 7-phosphate represents a glycolysis-independent entry and exit point into/from the non-oxidative PPP. The formation of this metabolite connects the PPP with open chain and polyol sugar metabolism and bacterial lipopolysaccharide biosynthesis. These connections with both glycolysis, amino acid biosynthesis and open-chain sugar metabolism place the PPP central to the metabolic network. Moreover, its flux and regulation not only depend on, but also influence its neighbouring metabolic routes, which might explain in part the extensive regulation of this biochemical route, as detailed in later sections of this article.

Analysis in yeast and mammalian cells has shown that with the exception of RPI, most of the PPP enzymes are not essential for survival at the cellular level. At the organism level in mammals, at least partial deficiencies of PPP enzymes G6PDH, 6PGDH, TAL and RPI are viable as well, but lead to severe genetic disease (see Section VI). However, no disease phenotypes or deficiencies have been reported for the other PPP enzymes; most likely their deficiency is embryonically lethal to mammalian organisms, indicating that the PPP as pathway is essential. Indeed, double gene deletions that affect both the oxidative and the non-oxidative PPP are also lethal down to the cellular level (Schaaff-Gerstenschlager & Zimmermann, 1993; Juhnke *et al.,* 1996; Krüger *et al.,* 2011). The viability of the partial PPP deficiencies and several null alleles therefore indicates that the oxidative and non-oxidative branches of the PPP can work independently; each of the two parts can compensate and provide sufficient sugar phosphate precursors required for cellular survival.

### (1) The L-type PPP and alternative or extended reaction sequences of the PPP

The PPP might also exist in alternative reaction sequences. Named the L-type PPP, a reaction sequence was proposed in liver cells that involves flux over alternative metabolites such as arabinose 5-phosphate and glycero-ido-octulose phosphate (Williams *et al.,* 1984). Also, alternative seven-carbon phosphates and their diphosphates have been associated with the PPP (Wood, 1985). (Longenecker & Williams, 1980) suggested that up to 30% of the PPP flux in hepatocytes could be attributable to these alternative PPP forms. Nevertheless, there is only limited confirmation of these PPP alternatives, indeed biochemical evidence for them has been questioned (Landau & Wood, 1983). Therefore, these alternatives are not addressed in detail herein. The recent discovery of the PPP enzymes SHPK and the SH17BPase however indicates that the full biochemical spectra of the PPP could exceed the core reactivity of the canonical pathway (Fig. 1), and hence that additional discoveries might still be made.

### (2) The subcellular localization of the PPP and its enzymes

In most organisms, including fungi and metazoa, the PPP is localized in the cytosol, and contributes both to the cytoplasmic metabolite as well as redox cofactor pool. However, important exceptions do exist. The pathway is split between the cytosol and other organelles such as the plastid, peroxisomes or glycosomes in plants and parasitic protozoa, respectively (Zimmer, 2001; Hannaert *et al.,* 2003; Kruger & von Schaewen, 2003). Part of the PPP might occur in the endoplasmic reticulum (ER) too. Microsomes, vesicles formed from the ER when cells are mechanically homogenized, contain at least five PPP enzymes. These include hexose 6-phosphate dehydrogenase (H6PDH), an enzyme similar to G6PDH (Bublitz & Steavenson, 1988; Nelson, Lehninger & Cox, 2008; Senesi *et al.,* 2010) that is required to provide NADPH to the luminal reductases (Beutler & Morrison, 1967; Takahashi & Hori, 1978; Senesi *et al.,* 2010). H6PDH has a broader range of substrates than G6PDH and it was described as being non-selective regarding the nucleotide cofactor (NAD^+^ and NADP^+^). The concentration of reduced NADP(H) in the endoplasmic lumen suggested that under physiological conditions glucose 6-phosphate and NADP^+^ are preferred. Hence, while the PPP is largely a cytosolic pathway, alternative organelle localisations do exist and are of significant importance.

### (3) Glucose 6-phosphate dehydrogenase (G6PDH) and the role of the oxidative PPP in NADPH synthesis

The most intensively studied enzyme of the PPP is G6PDH, an NADP^+^-dependent oxidoreductase. This enzyme has often been quoted as being rate limiting for the oxidative branch of the PPP. Although the classic concept of ‘rate limitation’ has its limitations (Kacser, 1995), the first enzymatic step involving G6PDH is certainly of central importance as the oxidative PPP is largely considered unidirectional. Eukaryotic G6PDH was first discovered in different strains of brewery yeast (Dickens, 1938), and to date this model organism has served for dissecting most of the functionality of the PPP. Budding yeast G6PDH is encoded by a single gene YNL241C (Nogae & Johnston, 1990; Thomas, Cherest & Surdin-Kerjan, 1991). Deletion of this gene retains viability, but *zwf1* cells are unable to synthesize methionine. It is assumed that this methionine auxotrophy is a consequence of the insufficient production of NADPH to sustain methionine biosynthesis, and requires yeast to assimilate ‘inorganic sulphur’ in order to form ‘organic sulphur’ (methionine or cysteine) to grow (Masselot & De Robichon-Szulmajster, 1975; Nogae & Johnston, 1990; Thomas *et al.,* 1991). This notion of NADPH shortage in *zwf1*Δ cells is supported by the observations that (*i*) when supplying NADPH from a different source, i.e. through alcohol dehydrogenase (Ald6), the methionine prototrophy is restored (Grabowska & Chelstowska, 2003). Moreover (*ii*), also yeast cells deleted for cytoplasmic superoxide dismutase (*SOD1*) become methionine auxotrophs (Slekar, Kosman & Culotta, 1996). These results indicate that G6PDH, and the oxidative PPP in general, play a quantitative role in NADP^+^ to NADPH recycling and redox balancing.

The importance of the NADPH-producing function of the PPP has been corroborated in several studies mainly addressing the antioxidant function of this coenzyme in yeast and mammalian cells. As NADPH is required as a redox equivalent in the antioxidant machinery, involving the thioredoxin/peroxiredoxin and glutathione systems (Pollak, Dölle & Ziegler, 2007*a*; Grant, 2008), yeast and mammalian cells deficient for G6PDH become hypersensitive to several oxidants (Juhnke *et al.,* 1996; Gorsich *et al.,* 2006; Krüger *et al.,* 2011).

Which proportion of the cytoplasmic NADPH pool is derived from the PPP? It varies, as the activity of the oxidative PPP is flexibly regulated, and as discussed in *Section III*, is actively increased during stress situations. A flexible flux of the PPP is supported from studies of NADPH-consuming enzymes, metabolic flux analysis, but in particular by investigations on the oxidative stress response. An illustrative example concerns the yeast NADPH oxidase *YNO1*, a recently discovered enzyme that similar to mammalian NADPH oxidases, oxidizes NADPH to produce superoxide. When *YNO1* is overexpressed in wild-type cells, superoxide levels increase 10-fold. An increase in superoxide levels is however no longer observed upon deletion of *zwf1*, indicating that the oxidative PPP compensates for the increased NADPH consumption caused by the *YNO1* overexpression (Rinnerthaler *et al.*, 2012).

Yeast cells deficient in NADPH production due to *zwf1* deletion have an almost normal NADPH/NADP^+^ ratio when growing exponentially and in glucose media. Their NADPH/NADP^+^ ratio however collapses when exposed to oxidants (Castegna *et al.,* 2011). Thus, the contribution of the oxidative PPP to the cellular NADPH pool is dynamic and context dependent, and essential for most cell types only when the NADPH requirement is increased. In Section IV we discuss mechanisms that facilitate a dynamic control of PPP activity under different physiological conditions, which is achieved through cooperation of transcriptional regulation, post-translational modifications, and allosteric control (feedback and feedforward regulation) of the involved enzymes.

In mammalian cells, G6PDH was intensively studied because partial deficiency in this enzyme represents the most common human enzyme defect, and as described in Section VI, has severe haematological consequences (haemolytic anaemia). A full depletion of G6PDH in mammals and nematodes is however lethal at the organism level (embryonic lethality) (Longo *et al.,* 2002; Ying, 2007) while the same mutation is tolerated at the cellular level (Pandolfi *et al.,* 1995). Similar to yeast cells, mouse embryonic stem cells possessing a mutation leading to a strong reduction in G6PDH activity are able to grow but are sensitive to externally applied oxidative stress (Pandolfi *et al.,* 1995; Filosa *et al.,* 2003). Also, mouse fibroblasts carrying a permanent deletion of the G6PDH exon are viable, despite their low clonogenicity (Filosa *et al.,* 2003).

#### (a) Non-PPP sources of NADP(H)

The role of the PPP in providing NADPH has to be seen in the context of other NADPH oxidoreductases, cellular compartmentalisation and the NAD(H)/NADP(H) *de novo* synthesis pathways. In many cell types and most conditions, NADP(H) is present mostly in its reduced form (Ying, 2007; Pollak *et al.,* 2007*a*). However, this assumption has a degree of uncertainty.

As membranes are considered to be NADPH impermeable, the NADPH recycling process and *de novo* biosynthesis is compartment-specific (Ying, 2007; Pollak, Niere & Ziegler, 2007*b*). Hence, in most organisms the PPP contributes mainly to the cytoplasmic NADPH pool. In mammalian mature erythrocytes which have no nucleus and no mitochondria, the PPP is generally assumed to be the dominating source of this coenzyme. In other cell types, there are important additional cytoplasmic enzymes that contribute to the NADPH pool, including the cytosolic isoforms of isocitrate dehydrogenase, glutamate dehydrogenase, methylene-tetrahydrofolate dehydrogenase, formyl-tetrahydrofolate dehydrogenase, aldehyde dehydrogenase and malic enzyme (Bernt & Bergmeyer, 1974; Wermuth, Münch & von Wartburg, 1977; Scheibe, 1987; Lee *et al.,* 2002; Fan *et al.,* 2014). Another source influencing the NADPH level in mammalian cells, for instance in mitochondria, appears to be trans-hydrogenation between NADH and NADP^+^, forming NAD^+^ and NADPH (Jackson, 2003; Venditti, Napolitano & Di Meo, 2013). The enzyme catalysing this reaction, nicotinamide nucleotide trans-hydrogenase, is an energy-driven integral protein of the inner mitochondrial membrane, and required in mitochondria to maintain their high NADPH/NADP^+^ ratio (Ronchi *et al.,* 2013).

Finally, in the debate about NADPH sources its *de novo* synthesis is less often taken into account. The synthesis of NADPH *de novo* is achieved by phosphorylation of NAD(H) by NAD kinase enzymes (Bieganowski *et al.,* 2006; Pollak *et al.,* 2007*a*). The lack of an NADP(H) phosphatase in many organisms implies that the *de novo* synthesis might primarily be used for the initial synthesis of the NADP(H) molecules, and not necessarily for controlling the NADP^+^/NADPH balance. Nonetheless it remains plausible that certain cells might be able to compensate for a lack of NADPH by *de novo* synthesis of the reduced form by phosphorylation of NADH.

#### (b) The synthesis of ribulose 5-phosphate in the non-oxidative PPP

**Fig. 2:**
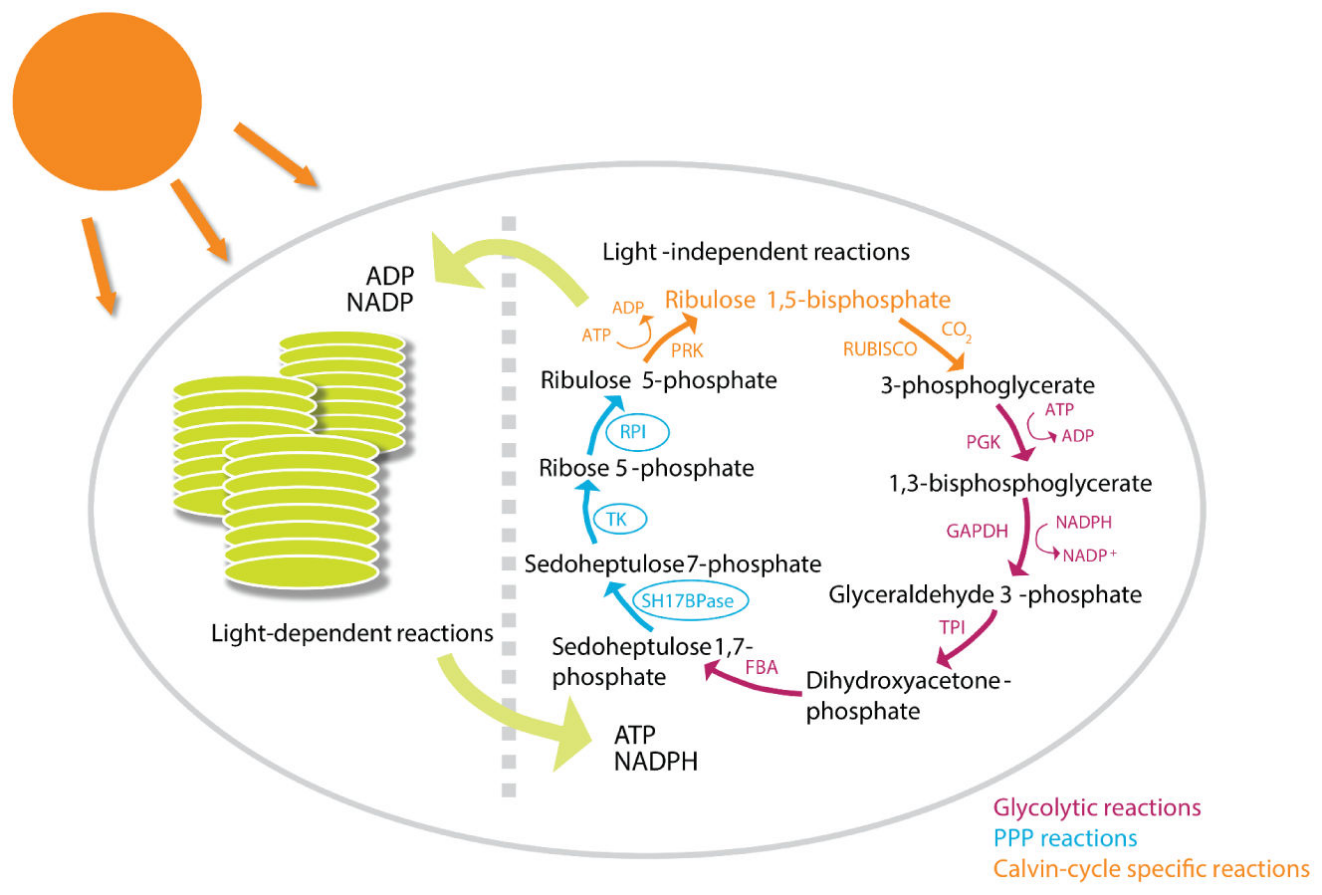
The light-independent reactions of carbon fixation in the Calvin cycle share enzymes and reactions with the pentose phosphate pathway (PPP) and glycolysis. Abbreviations are defined in Table 1; PGK, phosphoglycerate kinase; PRK, phosphoribulokinase, TK, transketolase; FBA, fructose-bisphosphate aldolase.

The pentose phosphate pathway in yeast and mammals shares much with the most important carbon assimilatory pathway in plants, the Calvin cycle. Reverse flux through the complete PPP could in theory assimilate carbon in a cyclic manner. The problem is that certain reactions of the oxidative PPP are not readily reversible. Accordingly, the Calvin cycle bypasses these reactions *via* ribulose 1,5-bisphosphate carboxylase oxygenase (Rubisco), apparently the most abundant metabolic enzyme in the biosphere (Raines, 2003). Rubisco converts ribulose 1,5-bisphosphate plus carbon dioxide into two molecules of 3-phosphoglycerate. While this enzyme is not shared with the PPP, other Calvin cycle reactions are (Fig. 2).

In particular, both the non-oxidative PPP and the Calvin cycle interconvert a total of 15 pentose carbon atoms (contained in ribulose 5-phosphate) with 15 glycolytic carbon atoms (in the form of fructose 6-phosphate and glyceraldehyde 3-phosphate), sharing some important reactions. However, while the classical non-oxidative PPP uses TAL to make sedoheptulose 7-phosphate, the Calvin cycle uses the glycolytic enzyme fructose-bisphosphate aldolase (FBA) to convert erythrose 4-phosphate plus dihydroxyacetone phosphate into sedoheptulose 1,7-bisphosphate, which in turn is hydrolysed by the enzyme SH17BPase to yield sedoheptulose 7-phosphate. This hydrolysis step provides the thermodynamic driving force, pushing the Calvin cycle towards ribulose 5-phosphate. Thus, while the non-oxidative PPP is reversible, the Calvin cycle is not.

Because FBA is a ubiquitous enzyme (playing an essential role in glycolysis and gluconeogenesis, and also producing sedoheptulose 1,7-bisphosphate), the distinguishing enzyme of the Calvin cycle’s path from triose phosphates to pentose phosphates is SH17BPase. Until recently, this enzymatic activity was thought to be specific to photosynthetic organisms. Metabolomic screening of yeast strains lacking genes of unknown function, however, revealed a strain with elevated sedoheptulose 1,7-bisphosphate. The associated gene was subsequently shown to encode an enzyme with SH17BPase activity involved in a novel variant of the non-oxidative PPP that follows yet more closely the Calvin cycle reaction sequences (Clasquin *et al.,* 2011). This thermodynamically driven variant of the non-oxidative PPP is termed riboneogenesis. Just as gluconeogenesis uses the energy of a sugar phosphate bond to convert trioses into hexoses, riboneogenesis uses one to drive flux from trioses to pentoses.

Ribose 5-phosphate biosynthesis *via* riboneogenesis is useful when demand for ribose exceeds that for NADPH. In such cases it is presumably advantageous to have a thermodynamically driven alternative to the standard non-oxidative PPP, and to avoid an over-reduction of the NADPH pool. Evidence for this effect was provided by experiments in yeast (Clasquin *et al.,* 2011). The cells were fed with glucose labelled selectively at the 6-position with carbon 13 (6-^13^C-glucose). Such glucose produces doubly labelled sedoheptulose 7-phosphate selectively *via* SH17BPase. This labelling pattern was observed preferentially when yeast cells were grown on media that decreased their need for NADPH (e.g. by providing them with lipids). One can envision the possibility that growing mammalian cells, including cancer cells, could also in some circumstances need ribose 5-phosphate in excess of NADPH, i.e. when DNA and RNA nucleotide synthesis is maximized (Ferreira, 2010; Cairns *et al.,* 2011). So far, however, has not been observed in doubly labelled sedoheptulose 7-phosphate 6-^13^C-glucose in mammalian cells (J. D. Rabinowitz, unpublished results). Thus, SH17BPase activity plays a role in plant and microbial metabolism, but not necessarily in animals.

In mammalian cells, a different additional influx into the sedoheptulose 7-phosphate PPP has been discovered recently: SHPK. This enzyme was identified based on the observation that several patients suffering from nephropathic cystinosis (CTNS) possess elevated urinary concentrations of sedoheptulose. In these patients, the CTNS gene was lost due to a 57 kb deletion, which aside from the CTNS gene also contained a gene encoding for a carbohydrate kinase-like (CARKL) protein. Biochemical assays have then shown that CARKL is in fact a sedoheptulokinase (SHPK) and catalyses the ATP-dependent phosphorylation of sedoheptulose (Kardon *et al.,* 2008; Wamelink *et al.,* 2008*b*). Apparently, the existence of SHPK implies that mammalian cells are able to convert sedoheptulose, and thus non-phosphorylated sugars, into ribose 5-phosphate and glycolytic intermediates. The role of SHPK could be to prevent an accumulation of sedoheptulose and related sugars in the clearance of polyol metabolites (Kardon *et al.,* 2008; Wamelink, Struys & Jakobs, 2008*a*). Moreover, expressing this gene in yeast increased H_2_O_2_-resistance, indicating that a second biological role of SHPK could consist of providing an increase of the PPP flux during the oxidative stress (Krüger *et al.,* 2011). Finally, as discussed in Section VII, SHPK could also ‘report’ altered metabolism to the immune system; expression of this gene directs macrophage polarization through control of glucose metabolism (Haschemi *et al.,* 2012).

## III. THE GLYCOLYSIS/PPP TRANSITION: METABOLIC AND TRANSCRIPTIONAL MECHANISMS THAT CHANGE PPP FLUX UPON DEMAND

The survival of a cell in its ever-changing environment depends on the robustness, interconnection and functionality of its biological networks. These are highly dynamic and respond to changing endogenous and exogenous conditions by interactions of a specific and limited set of components (Ihmels, Levy & Barkai, 2004; Ralser *et al.,* 2007; Chechik *et al.,* 2008; Buescher *et al.,* 2010; Fendt *et al.,* 2010; Grüning, Lehrach & Ralser, 2010). Such dynamic activity is particularly relevant for the metabolic network, where a few hundred metabolites are interconnected through biochemical reactions within metabolic modules, providing energy and biomolecules depending on substrate availabilities, enzyme activities and cellular demands. Therefore, to ensure proper functionality of the metabolic network upon environmental changes, metabolism is adapted. These adaptations involve the production of increased amounts of components needed and decreased concentrations of those unneeded, to save resources and energy simultaneously, and importantly, maintain homeostasis and prevent a collapse of the metabolic network. Moreover, these reconfigurations are highly regulated ensuring that concentrations of general cofactor metabolites, such as NAD(H), NADP(H) and A(T)P are not falling to fatal levels, the flux of the metabolic network is stabilized, and enzyme activity and abundance of the metabolic module is adjusted (Ihmels *et al.,* 2004; Patil & Nielsen, 2005; Cakir *et al.,* 2006; Ralser *et al.,* 2009; Grüning *et al.,* 2010; Heinemann & Sauer, 2010).

### (1) Regulation of the PPP during the oxidative stress response

**Fig. 3:**
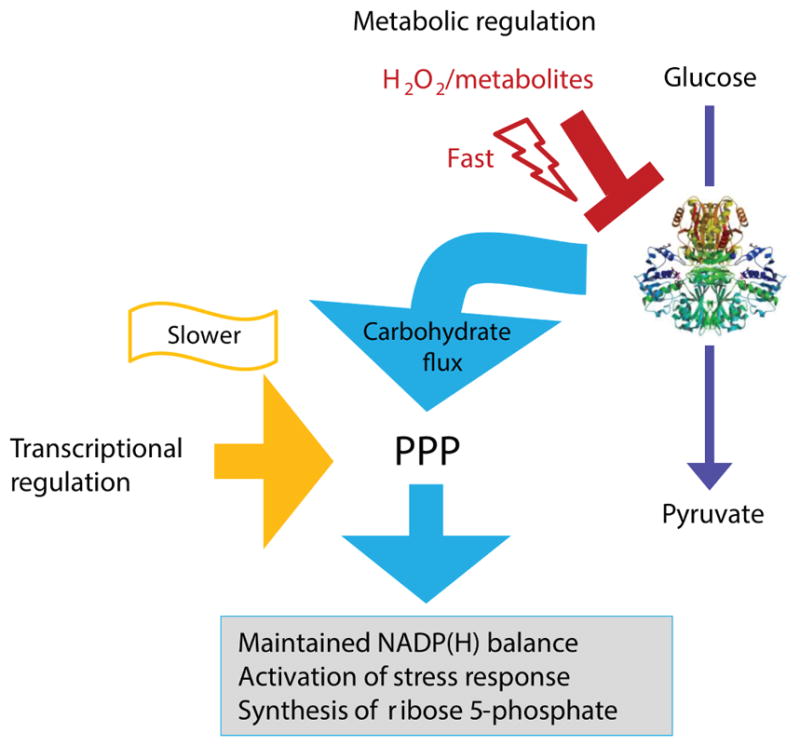
Induction of the glycolysis/pentose phosphate pathway (PPP) transition during oxidative stress. The PPP plays a pivotal role in counteracting oxidative stress and is implicated in (*i*) maintaining metabolic and redox homeostasis *via* NADP^+^ to NADPH reduction, (*ii*) by synthesizing ribose 5-phosphate used in nucleotide biosynthesis (increased synthesis is required upon DNA damage stress), and (*iii*) an important role in activating stress-responsive gene expression. In a stress situation, activity of the PPP is increased through orchestrated allosteric/post-translational (=metabolic) and transcriptional regulation, but these are not necessarily acting at the same time. The fastest response (~seconds timescale) is made possible through oxidative inhibition of glycolytic enzymes represented by the arrow moving from glyceraldehyde 3-phosphate dehydrogenase (GAPDH) (illustrated as a crystallographic structure), which acts as one of the metabolic switches, while the PPP remains active. This process is supported by post-translational modifications that increase glucose 6-phosphate dehydrogenase (G6PDH) activity. The comparatively slower (=minutes) process of altering transcript and protein levels allows for cellular adaptation to stress in the long(er)-term response. The GAPDH crystallographic structure was obtained from RCSB-PDB (www.rcsb.org). PDB ID 3PYM: (DOI:10.2210/pdb3pym/pdb).

A paradigm example to study the rapid metabolic as well as transcriptional regulation of the metabolic network is the response of the PPP to oxidative stress. As aforementioned, in yeast the NADPH-producing role of G6PDH is compensated by other NADP-oxidizing enzymes under normal growth conditions. However the NADP^+^/NADPH ratio collapses upon a hydrogen peroxide (H_2_O_2_) exposure, rendering G6PDH null cells highly oxidant sensitive (Nogae & Johnston, 1990; Todisco *et al.,* 2006; Castegna *et al.,* 2010). Indeed, the activity of the PPP is rapidly augmented when cells are exposed to the oxidant. To induce this metabolic transition, metabolic and gene regulatory mechanisms cooperate (Fig. 3). In the first seconds upon an oxidative burst, enzymes of glycolysis, glyceraldehyde 3-phosphate dehydrogenase (GAPDH) (Ralser *et al.,* 2007) and pyruvate kinase (PK) (Anastasiou *et al.,* 2011; Grüning *et al.,* 2011) are inactivated causing a block in glycolysis, while the flux of the PPP continues (Shenton & Grant, 2003; Ralser *et al.,* 2007; Ralser *et al.,* 2009). This rapid response lasts a few seconds to minutes, then transcriptional responses take over and maintain higher PPP activity through up-regulation of enzymes and post-translational modifications, including those which increase the activity of G6PDH (Chechik *et al.,* 2008; Ralser *et al.,* 2009; Cosentino, Grieco & Costanzo, 2011; Wang *et al.,* 2014). This tight regulation seems to have a dual role. During normal growth, it prevents an overproduction of NADPH and PPP intermediates, and minimizes carbon depletion due to CO_2_ production. At the same time, it facilitates a rapid cellular response when stress conditions apply (Shenton & Grant, 2003; Ralser *et al.,* 2007; Ralser *et al.,* 2009; Grant, 2008).

The temporal inhibition of glycolysis to the benefit of the PPP flux appears to be dependent on different mechanisms. GAPDH for instance is rapidly inactivated by chemical oxidation which correlates well with a boost in PPP metabolite concentrations observed within a few seconds (Ralser *et al.,* 2007; Ralser *et al.,* 2009). Other mechanisms that support the inhibition of glycolytic enzymes concerns allosteric control. A higher activity of the PPP is maintained by feedback inhibition of triosephosphate isomerase (TPI) by the glycolytic intermediate phosphoenolpyruvate (PEP) (Grüning *et al.,* 2014). PEP is the substrate of pyruvate kinase, that itself is controlled allosterically (Lyssiotis *et al.,* 2012; Morgan *et al.,* 2013). A third strategy that facilitates rapid PPP activation appear to be post-translational modifications which affect the activity of G6PDH. In mammalian and *Xenopus laevis* cells phosphorylation and acetylation increase G6PDH activity during the stress response, so that this enzyme does not become rate limiting (Cosentino *et al.,* 2011; Wang *et al.,* 2014).

The glycolytic/PPP transition during oxidative stress is mechanistically related to steady-state adaptation to physiological conditions that are associated with increased reactive oxygen species (ROS) production. Here, PK and its feedback regulatory function on TPI and other metabolic enzymes play a crucial regulatory role. In budding yeast, the activity of PK is reduced when cells respire at high rate, and less active isoforms (i.e. *PKM2* in mammals, *PYK2* in yeast) are expressed. The resultant accumulation of PEP causes feedback inhibition of several glycolytic enzymes, including the redox regulator TPI, and flux in the PPP increases (Grüning *et al.,* 2011; Grüning *et al.,* 2014). TPI inhibition by PEP was required to prevent oxidative stress and oxidative damage, and led to protein oxidation and mitochondrial damage in respiring cells when interrupted (Grüning *et al.,* 2011; Grüning *et al.,* 2014). As described in Section VIII, a similar mechanism appears to be used by cancer cells to maintain their metabolic redox balance as well.

### (2) Transcriptional regulators of the PPP

The concerted allosteric/post-translational response is followed by transcriptional events and transcripts and proteins of the PPP increase in concentration (Chechik *et al.,* 2008). The transcriptional changes occur in a fully coordinated manner, and enzymes are subsequently induced depending on their molecular function (Ihmels *et al.,* 2004; Chechik *et al.,* 2008). Therefore, the strictly timed program facilitates the cell’s reaction against minatory redox collapse immediately *via* the metabolome and the proteome, then later *via* the transcriptome, to adapt to the cellular responsibilities (Fig. 3). Such transcriptional patterns shape metabolic network gene regulation in response to changing conditions due to co-expression of enzymes that catalyse connected reactions.

The details of transcriptional regulation of PPP enzymes varies strongly among organisms; therefore, only principal mechanisms will be discussed here. Both in mouse and yeast, G6PDH is transcriptionally induced upon oxidative stress, and by the need for NADPH and PPP intermediates for anabolic reactions such as lipid synthesis and nucleotide synthesis (Kletzien, Harris & Foellmi, 1994; Lee *et al.,* 1999; Stanton, 2012). These effects are not specific to G6PDH, other PPP enzymes are dependent on transcriptional mechanisms as well (Kletzien *et al.,* 1994; Lee *et al.,* 1999; Stanton, 2012). This transcriptional regulators differs according to the specific demands of the cell or tissue. For instance, PPP regulation for lipid synthesis is achieved by the sterol regulatory element-binding proteins (SREBPs) class transcription factors, whereas the regulation during oxidative stress is mediated by nuclear respiratory factor 2 (Nrf 2)-family and other transcription factors. The latter also govern synthesis of many enzymes directly involved in oxidative stress defence (Stanton, 2012). In budding yeast, PPP gene expression control during oxidative stress is also exerted by basic leucine zipper (bZIP, Yap1) transcription factors and the nuclear response regulator Skn7. These factors, acting either in concert or as single regulators, govern the cellular response not only to oxidative stress, but also when anabolic intermediates are needed (Lee *et al.,* 1999). During oxidative stress, another regulatory role has been attributed to the transcription factor *Sin Three Binding protein 5* (Stb5), which activates PPP enzymes in response to exposure to the thiol oxidizing agent diamide (Akache, Wu & Turcotte, 2001; Larochelle *et al.,* 2006; Hector *et al.,* 2009).

### (3) Feedforward regulation of the metabolome to the transcriptome: PPP metabolites as regulators of the stress response

During stress conditions, the PPP seems to have attained another role: the induction of stress-responsive gene expression. Evidence for an NADPH-independent function of the PPP in the antioxidant response comes from the observation that enzyme deficiencies of both PPP branches are oxidant sensitive (Juhnke *et al.,* 1996; Krüger *et al.,* 2011). Moreover, a yeast double mutant deleted for G6PDH (Zwf1) and the non-oxidative PPP enzyme Tal1 is more H_2_O_2_ sensitive than the parent mutants deleted for either Tal1 or Zwf1 alone (Krüger *et al.,* 2011). By contrast, increased oxidant resistance was obtained when the metabolite load of the non-oxidative pathway was augmented due to expression of the mammalian SHPK in yeast (Kardon *et al.,* 2008; Krüger *et al.,* 2011). In addition, stress response genes were induced when the flux of the PPP was stimulated by genetic perturbation of glycolysis (Krüger *et al.,* 2011). Finally, tuning the NADPH demand gradually by overexpression of an engineered NADPH-dependent butanediol dehydrogenase led to a concomitant accumulation of PPP metabolites and also triggered the induction of PPP and stress response genes (Celton *et al.,* 2012). Hence, during the stress response, the PPP appears to play not only the role of a canonical metabolic pathway which *responds* to oxidant treatments, but also functions as a transcriptional balancer and is involved in *inducing* components of the oxidative stress response. The exact underlying molecular mechanisms are however yet unknown.

## IV. ANALYTICAL METHODS FOR MEASUREMENT OF PPP INTERMEDIATES

Key to finding new PPP reactions, as well as elucidating regulation of the pathway, are reliable methods to quantify PPP flux and intermediate concentrations. Major challenges of studying the PPP include the high turnover rates of its intermediates in the second or sub-second time range (Weibel, Mor & Fiechter, 1974; De Koning & van Dam, 1992; Douma *et al.,* 2010) and low abundances of these compounds (Casazza & Veech, 1986). Rapid sampling techniques such as cold methanol quenching are typically employed to arrest metabolism immediately in cells (De Koning & van Dam, 1992). A further difficulty in sugar phosphate analytics is the proper separation of pentose isomers (ribose 5-phosphate, ribulose 5-phosphate and xylulose-5-phosphate) and hexose isomers (glucose 6-phosphate, fructose 6-phosphate and other relevant hexose monophosphates).

### (1) From historical techniques to LC-MS/MS

A series of different methods have been developed to study PPP metabolites and enzymes, including colorimetric assays (Sable, 1952; Novello & McLean, 1968) and the use of thin layer chromatography in combination with ^14^C-labelled substrates (Becker, 1976). Another widely used quantification approach is to couple the enzymatic interconversion of specific sugar phosphate substrates to the NAD-dependent oxidation of glyceraldehyde 3-phosphate by GAPDH, or other reactions catalysed by NADP(H)- or NAD(H)-dependent enzymes, and to monitor the consumption of NAD(P)H by spectrophotometric or fluorometric methods (Sable, 1952; Kauffman *et al.,* 1969; Casazza & Veech, 1986; King, Passonneau & Veech, 1990). However, these procedures are limited to measuring one component at a time and are critically dependent on the specificity of purified enzymes and optimal assay conditions. Detailed studies of the PPP were therefore accompanied by long, cumbersome analytical methods with relatively low sensitivity and virtually no dynamic over time (Casazza & Veech, 1986).

With the appearance of high-performance liquid chromatography (HPLC) these extensive measurement times could be drastically reduced to 30–180 min (Giersch, 1979; Smrcka & Jensen, 1988; Swezey, 1995), giving rise to major advances in the field of PPP research. A combination of chromatographic methods with mass spectrometry eventually facilitated the routine separation and analysis of sugar phosphates. Several capillary electrophoresis-MS (Soga, 2007) and gas chromatography-MS methods (Koek *et al.,* 2006; Cipollina *et al.,* 2009) have been developed; however, they have already been relatively outnumbered by a number of liquid chromatography tandem mass spectrometry (LC-MS/MS) techniques for sugar phosphate measurement. In a targeted LC-MS/MS approach, Wamelink *et al.,* 2005 determined absolute concentrations of a series of sugar phosphate intermediates by means of HPLC and tandem mass spectrometry. Later, analogous methods were used to measure extended sets of metabolites (Luo *et al.,* 2007; Buescher *et al.,* 2010; Jannasch, Sedlak & Adamec, 2011; Rühl *et al.,* 2012; Lu *et al.,* 2010; Bajad *et al.,* 2006). Without doubt, mass spectrometry as the detection system has strongly enhanced sugar phosphate analysis; but difficulties in separating structural isomers still need to be overcome. The development therefore of further powerful separation procedures will be of high importance to allow for a more reliable and robust quantification of PPP metabolites.

In addition to measuring metabolite levels, there has been long-standing interest in measuring PPP flux. One classical and reliable approach to measuring absolute oxPPP flux in cells involves feeding, in separate experiments, 1-^14^C-glucose and 6-^14^C-glucose and measuring radioactive CO_2_ release (Katz & Wood, 1963). As carbon 1 of glucose is selectively released by the oxPPP whereas other pathways metabolize carbon 1 and 6 identically, the difference in radioactive CO_2_ release from these two tracers provides direct quantitation of the oxPPP flux. Kinetic analysis of PPP intermediate labeling from ^13^C-glucose by LC–MS can also be used to calculate absolute oxPPP flux and gives similar estimates to the ^14^C-CO_2_-release approach, but the ^14^C-approach remains more precise (Fan *et al.,* 2014).

A strategy based on the cleavage of carbon 1 of glucose by the oxPPP has also been employed to measure oxPPP relative to non-oxPPP flux into ribose-5-phosphate. One method involves feeding, separate experiments, 1-^13^C-glucose and 6-^13^C-glucose and measuring ribose-5-phosphate labeling by mass spectrometry, with ribose-5-phosphate produced via oxPPP labeled by 6-^13^C but not 1-^13^C-glucose. More conveniently, one can feed 1,2-^13^C-glucose and to look for singly versus doubly labeled ribose-5-phosphate, with the former made by the oxPPP and the latter by the non-oxPPP (Lee *et al.,* 1998). A limitation of these methods is that they do not distinguish between net ribose production by the non-oxPPP versus exchange flux (which can impact ribose-5-phosphate labeling even if net non-oxPPP flux is away from ribose). Thus, definitive methods for understanding non-oxPPP flux are still needed, and additional tracers and measurement of more metabolites’ labeling may, with proper computational deconvolution, provide further insights (Brekke *et al.,* 2012; Tang *et al.,* 2012; Crown *et al.,* 2012).

In this vein, recent work has provided a new tracer method for the PPP: Deuterium-labeled glucose (1-^2^H or 3-^2^H-glucose) to track specifically oxPPP-produced NADPH and its subsequent utilization for reductive biosynthesis (Fan *et al.,* 2014; Lewis *et al.,* 2014). Initial data show that the oxPPP accounts for about 50% of total NADPH in transformed mammalian cells growing in culture, with most of this NADPH devoted to fatty acid synthesis. These methods are now poised to quantitate variation in oxPPP activity and NADPH usage across conditions, cell types, and compartments.

### (2) *In vivo* PPP measurements using NMR

MS-based methods are sensitive, selective and robust, but are not applicable *in vivo*. Classic nuclear magnetic resonance (NMR) methods regularly fall short of providing the sensitivity required for studying the PPP. However, recently, a new NMR technique has been introduced, termed hyperpolarization, which can increase the sensitivity of the ^13^C NMR experiment by more than 10^4^-fold (Ardenkjaer-Larsen *et al.,* 2003). Being dynamic, this method could be used to measure PPP flux *in vivo*. A ^13^C-labelled cell substrate is mixed with a stable radical and cooled to temperatures close to absolute zero (~1 K) in a high magnetic field (typically 3.5-5T). At this temperature the electron spins in the radical are almost completely polarized. This polarization is then transferred to the ^13^C spins by microwave irradiation and the sample is then rapidly warmed to room temperature with substantial retention of the ^13^C spin polarization. Cells can then be exposed to the hyperpolarized ^13^C-labelled substrate, or for *in vivo* studies the tracer can be injected intravenously or added to the growth media of microorganisms. The signal is now boosted as a result of polarization of the ^13^C spins, so that the position of the molecule and the metabolites formed from it can be imaged (Brindle *et al.,* 2011; Kurhanewicz *et al.,* 2011).

The major limitation of the technique is the relatively short life time of the spin polarization (typically ~30 s *in vivo*), which means that only relatively rapid metabolic processes can be imaged and the experiment must be accomplished within 2–5 min following injection of the hyperpolarized substrate. Measurements with hyperpolarized [U-^2^H, U-^13^C] glucose in *E. coli*, yeast and breast cancer cells have shown production of hyperpolarized [1-^13^C] pyruvate or lactate, which allows real-time measurements of glycolytic flux (Meier, Jensen & Duus, 2011*a*; Meier *et al.,* 2011*b*; Harris, Degani & Frydman, 2013). The technique was recently translated to a clinical study of prostate cancer (Nelson *et al.*, 2013), and as discussed in Section VIII, is revealing the activity of the PPP in human cancer cell metabolism *in vivo*.

## V. THE PPP IN BIOTECHNOLOGY: METABOLIC ENGINEERING

The PPP is one of the most important targets for metabolic engineering and biotechnology. One way in which this pathway is utilized is as a source of NADPH and pentose sugars for the overproduction of various commercially and medically important compounds such as carotenoids (Schwender *et al.,* 1996; Martínez *et al.,* 2008), polymers (Kabir & Shimizu, 2003; Jung *et al.,* 2004), antibiotics (Jørgensen *et al.,* 1995; Avignone Rossa *et al.,* 2002; Butler *et al.,* 2002; Li & Townsend, 2006; Borodina *et al.,* 2008), alcohols (Jeppsson *et al.,* 2002; Jeffries & Jin, 2004; Hahn-Hägerdal *et al.,* 2007), nucleosides (Kamada *et al.,* 2001) and amino acids (Marx *et al.,* 1997; Herrmann & Weaver, 1999). Additionally, altering the PPP was used to prevent carbon exhaust during pentose fermentation (Verho *et al.,* 2002). Recently, the PPP has been utilized to create a synthetic non-oxidative glycolysis/PPP hybrid pathway able to produce energy significantly more efficiently by precluding carbon loss *via* carbon dioxide (Bogorad, Lin & Liao, 2013) – a proof of concept that metabolic engineering could contribute to reducing the current exhaust of greenhouse gases.

Focus on producing the biopolymer poly-hydroxybutyrate (PHB), a non-toxic biodegradable and bio-derived ‘green’ plastic (Hankermeyer & Tjeerdema, 1999), has included modification of both the oxidative and non-oxidative enzymes of the PPP. The insertion of *gnd* and *tktA* genes (6PGDH and TKL, respectively) from *E. coli* into the facultative chemolithoautotroph bacterium, *Ralstonia eutroph*a, amplified *gnd*, which overproduced NADPH, but also suppressed growth as well as PHB production. Conversely, amplification of *tktA* significantly increased the generation of PHB *via* efficient conversion of glyceraldehyde 3-phosphate into acetyl-coenzymeA, the precursor for PHB biosynthesis (Lee, Shin & Lee, 2003). Another attempt focused on generating PHB *via* the PPP targeted the oxidative pathway only. By deleting the *pgi* gene in *E. coli,* carbon flux was shown to be redirected through the PPP in turn increasing the production of NADPH, creating a reducing power imbalance and affecting cell growth. The introduction of the NADPH-consuming PHB biosynthetic pathway into the *pgi* knockout, allowed partial cell growth recovery (Kabir & Shimizu, 2003).

Another genus where modification of the PPP was successful in industrial application is *Streptomyces*, a workhorse for the generation of various antibiotics (Hopwood, 2007). To overproduce the pigmented antibiotics actinorhodin (ACT) and undecylprodigiosin (RED), the *pfkA2* gene was deleted in *S. coelicolor* A3(2), leading to increased flux through the PPP (Borodina *et al.,* 2008). Similarly, inactivation of the glycolytic genes *gap1* and *gap2,* encoding GAPDH, in *S. clavuligerus* was exploited to increase production of clavulanic acid, a *β*-lactamase inhibitor, used alongside penicillin and cephalosporin to combat antibiotic resistance (Li & Townsend, 2006). The overproduction of clavulanic acid was facilitated through increasing the supply of its precursor glyceraldehyde 3-phosphate. A recent study proposed that in order to increase the flux towards the PPP, TAL overexpression would be much more useful than GAPDH inactivation, because of the compromised carbon balance of the PPP (Linck *et al.,* 2014).

Modification of the PPP has also been effective in fungal biotechnology. The fungus *Penicillium chrysogenum* was exploited by enhancing flux through the PPP to increase NADPH levels, thereby increasing the penicillin yield (Jørgensen *et al.,* 1995). Other applications of the PPP in fungal biotechnology include the optimisation of alcohol, amino acids (e.g. lysine), nucleosides, inosine and 5′-xanthylic acid production (Marx *et al.,* 1997; Kamada *et al.,* 2001; Jeppsson *et al.,* 2002; Overkamp *et al.,* 2002; Verho *et al.,* 2002). Hence, in several instances an altered PPP flux was beneficial for biotechnological production cycles in both bacteria and yeast systems *via* its NADPH donor function, or inhibited to decrease carbon exhaustion. Thus, altering PPP activity is exploitable in both microbial and eukaryotic biotechnology in order to optimize cofactor- and sugar-phosphate-dependent processes.

## VI. INBORN ERRORS WITHIN PPP ENZYMES THAT LEAD TO HUMAN METABOLIC DISEASE

**Fig. 4:**
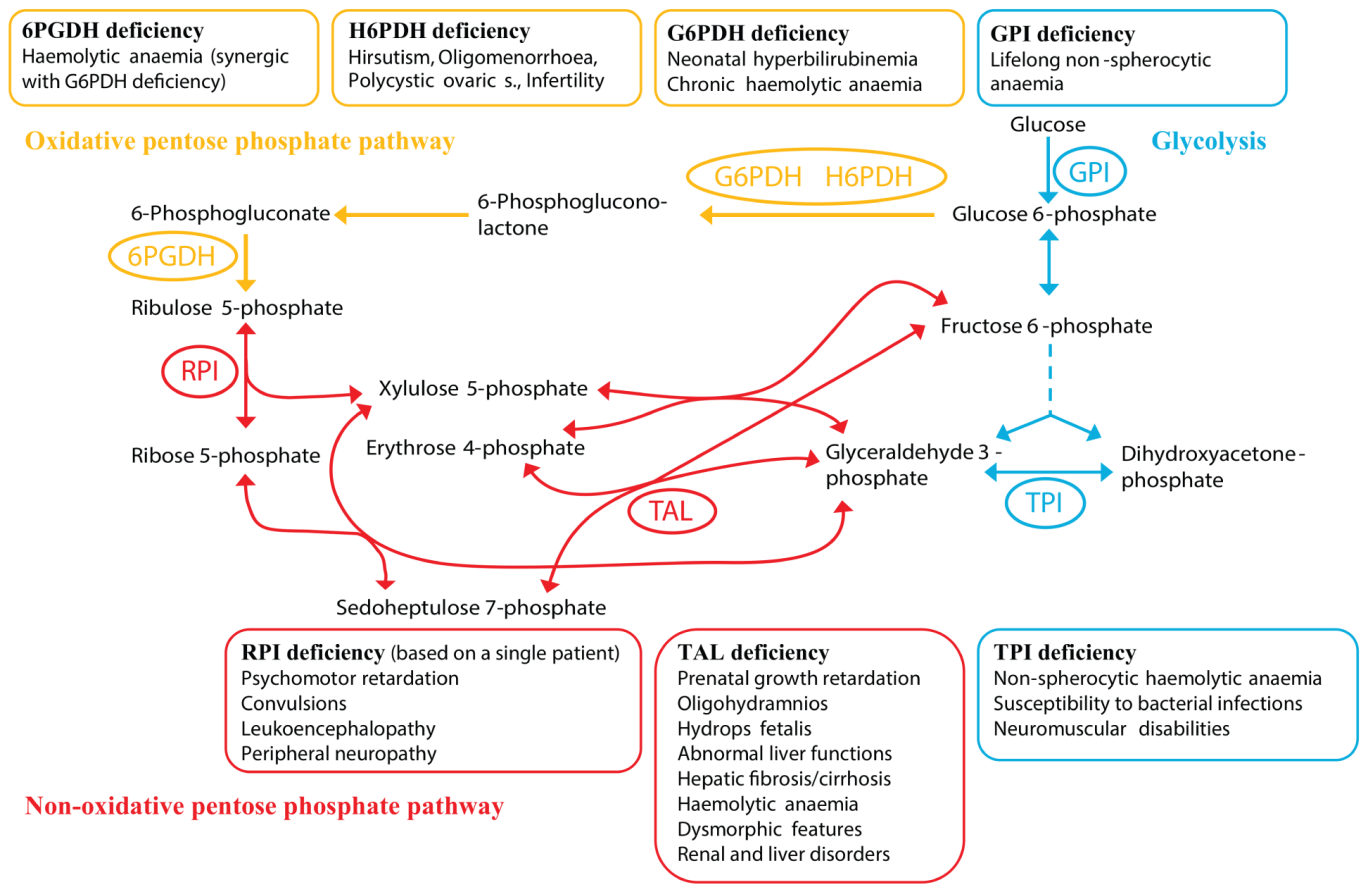
Inherited metabolic disease caused by pentose phosphate pathway (PPP) deficiencies, including two glycolytic enzymopathies with effects on the PPP. PPP enzymopathies are caused either by complete or partial deficiency of PPP and glycolytic enzymes. Abbreviations are defined in Table 1, H6PDH, hexose 6-phosphate dehydrogenase.

Four known metabolic genetic diseases are the direct consequence of a deficiency in a PPP enzyme; and at least two genetic disorders associated with the PPP are attributed to enzyme mutations in glycolysis *via* affecting PPP activity. Notably, these PPP disorders encompass both the most frequent human genetic defect (G6PDH deficiency) as well as the so-far rarest human disorder [ribose 5-phosphate isomerase (RPI) deficiency], where only a single patient has been diagnosed to date. The other defects, TAL deficiency, as well as the two glycolytic syndromes TPI and glucose phosphate isomerase (GPI) deficiency, occur at a different frequency but are considered rare disorders as well (Fig. 4).

### (1) G6PDH deficiency, the most common human enzyme defect

G6PDH deficiency (OMIM: 305900) is an X-linked disorder; the gene is located at the telomeric region of the long arm of the X chromosome (band Xq28) (Cappellini & Fiorelli, 2008; Van Zwieten, Verhoeven & Roos, 2014). Prevalent in more than 400 million people worldwide, it represents the most common heritable human enzyme defect (Cappellini & Fiorelli, 2008; Nkhoma *et al.,* 2009). The global occurrence of G6PDH deficiency is geographically correlated with areas inhabited by populations historically exposed to endemic malaria, including Africa, Mediterranean Europe, South-East Asia and Latin America (Ruwende & Hill, 1998).

The most frequent clinical manifestation is neonatal hyperbilirubinaemia and chronic haemolytic anaemia (Luzzatto & Mehta, 1995; Cappellini & Fiorelli, 2008; Van Zwieten *et al.,* 2014). The high frequency of the disorder is likely explained as reduced G6PDH activity appears protective against malaria caused by *Plasmodium falciparum* (Luzzatto & Bienzle, 1979; Ruwende & Hill, 1998). As the oxidative PPP is the only relevant NADPH source for red blood cells, a decrease in NADPH production is likely associated with the clinical phenotype, but potentially also explains this anti-malaria advantage. As a consequence, however, despite most carriers of mutant G6PDH alleles being asymptomatic, exposure to oxidative stressors such as artemisinin (and other drugs) or infections can elicit acute haemolysis in G6PDH patients. As such, the epidemiology of G6PDH deficiency has been related to the sickle cell anaemia phenotype, caused by Hbs and SS variants of haemoglobin. Sickle cell anaemia is associated with episodes of acute illness and progressive organ damage, but is also associated with heterozygous advantage against malaria (Rees, Williams & Gladwin, 2010).

G6PDH deficiency can be associated with a second, rare defect in the PPP, 6-phosphogluconate dehydrogenase (6PGDH) deficiency (Beutler, Kuhl & Gelbart, 1985). The first evidence of the enzyme deficiency was reported in 1963, when a female patient presenting G6PDH deficiency exhibited reduced activity of 6PGD as well (Brewer & Dern, 1964). More recently, also G6PDH independent incidences of this defect have been reported, and lead to reduced redox tolerance of erythrocytes (Caprari *et al.,* 2001).

### (2) RPI deficiency, the currently rarest human disorder

By contrast with G6PDH deficiency, other PPP disorders are exceptionally rare. Huck *et al.,* 2004 described a patient with a deficiency of RPI (OMIM: 608611) who suffered from leukoencephalopathy and peripheral neuropathy. This patient had psychomotor retardation from early in childhood and developed epilepsy at the age of four. From the age of 7 the patient experienced neurological regression, with deterioration of vision, speech, hand coordination, walking, and seizures. Neurological examination at the age of 14 years showed spasticity, bilateral optic atrophy, and nystagmus on lateral gaze, an increased masseter reflex and mixed cerebellar/pseudobulbar dysarthria. He had prominent cerebellar ataxia and mild peripheral neuropathy and displayed severe mental retardation. The patient is now (2014) in his twenties, and so far a unique case, as since the original report no further cases of RPI deficiency have been described.

The molecular diagnosis of the rare case of RPI deficiency was facilitated through a combination of metabolic profiling and candidate gene re-sequencing. Magnetic resonance imaging (MRI) is able to identify brain abnormalities in children with neurological deficits (Watkins, Gadian & Vargha-Khadem, 1999; Huck *et al.,* 2004). MRI of the patient showed extensive anomalies of the cerebral white matter with prominent involvement of the short association fibres (U-fibres), relative sparing of periventricular white matter, and complete sparing of corpus callosum and internal capsule (Van der Knaap *et al.,* 1999). Extremely high concentrations of pentitol metabolites (arabitol and ribitol) were found in the brain by magnetic resonance spectroscopy (MRS), and in cerebrospinal fluid, plasma and urine, as well as xylulose in the urine as tested by mass spectrometry (Van der Knaap *et al.,* 1999). These metabolites can derive from PPP intermediates xylulose, ribose 5-phosphate and ribulose 5-phosphate, which guided to the identification of the candidate gene by targeted re-sequencing of PPP enzymes. Two mutant alleles in the RPI encoding RPIA gene were demonstrated: a 1 bp deletion (540delG) resulting in a frameshift at codon 181 and a predicted truncated protein of 196 amino acids, and a missense mutation C182T, resulting in an Ala-to-Val substitution (A61V). The finding of two mutant alleles in the patient with apparently healthy parents suggests autosomal recessive inheritance. Genetic and biochemical evidence suggests an explanation for the rareness of the case: full RPI deficiency appears to be lethal. Studies of patient-derived cell lines and transgenic yeast models however revealed that the patient carried an uncommon allelic combination: he is heterozygous for a catalytically inactive RPI allele, whereas the second allele encodes a partially catalytically functional enzyme that exhibits a cell-type-dependent expression deficit in addition (Wamelink *et al.,* 2010).

### (3) TAL deficiency

Transaldolase deficiency (TAL or TALDO deficiency, OMIM: 606003) is caused by autosomal recessive deficiency in the human TAL-encoding gene (*TALDO1*) located on chromosome 11p15.5–p15.4, and has recently been diagnosed in more than 30 patients worldwide (Wamelink *et al.,* 2007; Wamelink *et al.,* 2008*a*; Tylki-Szymańska *et al.,* 2009; Balasubramaniam *et al.,* 2011; Eyaid *et al.,* 2013). TAL-deficient patients suffer from great phenotypic variability. Most patients display first symptoms in the neonatal or antenatal period, with prenatal intra-uterine growth retardation, oligohydramnios and hydrops foetalis being described (Valayannopoulos *et al.,* 2006). Newborns present with hepatosplenomegaly, bleeding diathesis, abnormal liver function, cholestatic jaundice and elevated liver enzymes, while in older patients, hepatic fibrosis or cirrhosis is the pathological liver hallmark. Most patients show haemolytic anaemia, dysmorphic features, neonatal oedema and congenital heart defects. Moreover, renal manifestations and endocrine disorders have been frequently reported (Loeffen *et al.,* 2012). Mild transient hypotonia was described in several patients but mental and motor development was normal in most patients. Recently, a TAL-deficient patient with early onset hepatocellular carcinoma with an 9 years old asymptomatic older brother were described (Leduc *et al.,* 2013).

TAL deficiency results in the accumulation of seven-carbon sugars (sedoheptulose, mannoheptulose), sedoheptulose 7-phosphate, and open chain sugar-alcohols (polyols) including erythritol, arabitol, ribitol, sedoheptitol and perseitol, and erythronic acid derived from the pathway intermediates (Verhoeven *et al.,* 2001; Wamelink *et al.,* 2005; Engelke *et al.,* 2010) that can help as biomarkers in diagnosis. The clinical picture of TAL deficiency is dominated by liver fibrosis/cirrhosis, resulting in permanent scar tissue. Since TAL has been recognized as a regulator of apoptotic signal-processing (Banki *et al.,* 1996), this might have relevance for the pathogenesis of liver disease, as observed in patients and in TAL-deficient mice (Perl *et al.,* 2011). In addition, accumulation of the metabolite sedoheptulose 7-phosphate has been suggested to be involved in the pathophysiology of liver cirrhosis (Verhoeven *et al.,* 2001), and could be functionally connected to the disease phenotype.

In a mouse model of TAL-deficiency, the accumulation of sedoheptulose 7-phosphate and a failure to recycle ribose 5-phosphate through the non-oxidative branch has been observed. Furthermore, diminished production of NADPH led to secondary depletion of reduced glutathione (GSH) and oxidative stress, as well as loss of the mitochondrial transmembrane potential and mitochondrial mass (Hanczko *et al.,* 2009). A decrease of NADPH was potentially caused by the conversion of five-carbon sugar phosphates to five-carbon polyols by aldose reductase at the expense of NADPH levels (Perl *et al.,* 2011). In some earlier diagnosed TAL-deficient patients, low levels of cholesterol, estradiol, testosterone or vitamin D were detected, indicating decreased NADPH/NADP^+^ and leading to decreased activity of NADPH-dependent reactions (i.e. cholesterol biosynthesis, hormone metabolism) (Banki *et al.,* 1996). Haemolytic anaemia was also observed in most patients, probably related to decreased NADPH production in erythrocytes as observed in G6PDH deficiency.

### (4) GPI deficiency

GPI catalyses the interconversion of glucose 6-phosphate to fructose 6-phosphate. A deficiency in this enzyme (OMIM: 613470) increases the flux in the PPP, as the glycolytic route of carbon metabolism becomes inhibited. Deficiency of erythrocyte GPI was first described in a boy with lifelong nonspherocytic anaemia in 1968 (Baughan *et al.,* 1968). In a patient diagnosed in 1985, the GPI deficiency syndrome was characterized by a deficiency in red cells, granulocytes and muscles (Schröter *et al.,* 1985). In 1993, another case of GPI deficiency was associated with hereditary nonspherocytic haemolytic anaemia (Shalev *et al.,* 1993). Mutations found in GPI deficiency retain residual activity of the enzyme, but the deficient enzymes were characterized by reduced thermostability (Kugler & Lakomek, 2000). The decreased activity of the isomerase causes an increase in glucose 6-phosphate, erythrose 4-phosphate and 6-phosphogluconate, indicating increased metabolite load and flux in the PPP.

In yeast cells grown on glucose, a full deficiency of GPI is lethal, but can be complemented by the overexpression of NADPH-oxidising enzymes. This indicates that the fatality of a full GPI deficiency results from redox cofactor imbalance due to NADPH overproduction in the PPP (Verho *et al.,* 2002).

### (5) TPI deficiency

TPI deficiency (OMIM: 615512) was one of the first enzymatic defects to be associated with the PPP. Schneider *et al.,* 1965 reported a deficiency of the enzyme in red blood cells referring to the disorder as Dacie’s type II haemolytic anaemia. TPI deficiency is further of historical importance in the treatment of rare diseases, as it was an early case where an enzyme replacement therapy was applied (Ationu *et al.,* 1999).

TPI deficiency is a rare and severe disease involving nonspherocytic haemolytic anaemia, leading to progressive neuronal degeneration, muscle degeneration and is associated with deadly infections and spasticity. In most cases, the affected children die before adulthood (Schneider, 2000; Orosz *et al.,* 2009). Since the discovery of the syndrome less than 100 patients have been diagnosed worldwide (Schneider & Cohen-Solal, 1996). This frequency is lower than the natural mutation rate would predict, but also lower as predicted from the estimated population frequencies of recessive TPI-deficient alleles. This indicates that homozygously defective alleles are embryonically lethal, a notion supported by studies in mice (Merkle & Pretsch, 1989). The substantial frequencies of heterozygote TPI deficiency lead to speculations of a heterozygous advantage of TPI- deficient alleles (Mohrenweiser, 1981; Mohrenweiser, Wurzinger & Neel, 1987; Watanabe, Zingg & Mohrenweiser, 1996). In a more recent study, the entire TPI locus was re-sequenced in 387 centenarians, and single nucleotide polymorphisms (SNPs) were genotyped in an even larger sample of long-lived individuals (*N* = 1422) and younger controls (*N* = 967). However, no heterozygous TPI deficient alleles were confirmed (Ralser *et al.,* 2008). The discrepancy could indicate that the observed differences in TPI activity had an epigenetic or post-translational cause, or that high frequencies of heterozygous TPI null alleles are a population-specific phenomenon.

Despite a substantial number of TPI- deficient alleles having been described (Schneider & Cohen-Solal, 1996), a single allele describes the majority of clinical cases. This allele carries a mutation exchanging a glutamic acid residue on position 105 (position 104 when not counting the ATG codon), to an aspartic acid, located in the region of the TPI enzyme responsible for dimer formation (Arya *et al.,* 1997; Schneider, 2000; Rodríguez-Almazán *et al.,* 2008). This allele has been the only one described to cause TPI deficiency in the homozygous state, and it was speculated that the allele may descend from a single individual that may have lived in what is now France or England around 1000 years ago (Arya *et al.,* 1997). Recently, the same allele has also been found in a Turkish family, but it is currently unclear whether it results from a *de novo* mutation (Sarper *et al.,* 2013). It has been discovered in a transgenic yeast model expressing the human isoform that this residue substantially interferes with the dimerisation of TPI, but does not *per se* interfere with catalysis (Ralser *et al.,* 2006). The global structure of TPI_E104D_ is similar to that of the wild-type; however, residue 104 is part of a conserved cavity that possesses an elaborate conserved network of buried water molecules at the dimer interface (Rodríguez-Almazán *et al.,* 2008). In the TPI_E104D_ mutant, a disruption of contacts of the amino acid side chains in the conserved cluster leads to a perturbation of the water network in which the water–protein and water–water interactions joining the two monomers are significantly weakened and diminished (Rodríguez-Almazán *et al.,* 2008). Hence, TPI deficiency is primarily caused by a structural defect.

How does TPI deficiency, a disorder caused by a structurally defective glycolytic enzyme, then relate to the PPP? In the course of generating a yeast model for TPI deficiency, it was discovered that TPI alleles with reduced catalytic activity render cells resistant to oxidants (Ralser *et al.,* 2006). In yeast, this was mainly described for thiol-oxidizing reagents such as diamide, however in *Caenorhabditis elegans* sensitivity was observed also for natural oxidants including juglone (Ralser *et al.,* 2007). Cells expressing the mutant TPI alleles possess increased concentration of PPP metabolites, and the antioxidant effects of TPI mutant alleles are fully dependent on the first enzyme of the oxidative PPP, G6PDH (Ralser *et al.,* 2007; Grüning *et al.,* 2011; Grüning *et al.,* 2014). In *Drosophila melanogaster*, the situation seems to be more complex and dependent on respiratory activity of the TPI mutant cells. Also *Drosophila melanogaster* cells’ TPI mutations affect oxidant resistance, however their redox status seem to shift towards oxidation (Hrizo *et al.,* 2013). Moreover, as shown below (Section VIII), TPI mutant alleles were important in understanding the role of the PPP’s antioxidant activity in cancer.

### (6) Hexose 6-phosphate dehydrogenase (H6PDH) deficiency

H6PDH is a luminal enzyme analogous to G6PDH responsible for NAD^+^ and NADP^+^ reduction in the endoplasmic reticulum. The enzyme oxidizes glucose 6-phosphate, glucose, galactose 6-phosphate and 2-deoxyglucose 6-phosphate (Krczal, Ritter & Kömpf, 1993; Senesi *et al.,* 2010). Several allelic variants of H6PDH mutations are known and result in hirsutism, oligomenorrhoea, obesity, acne and infertility (Jamieson *et al.,* 1999; Draper *et al.,* 2003; Lavery *et al.,* 2008). Draper *et al.,* 2003 hypothesized that mutations in H6PDH could cause an NADPH deficiency in the endoplasmic reticulum (ER), affecting the directionality of the 11-beta-hydroxysteroid dehydrogenase type 1 (HSD11B1) reaction. HSD11B1 is a regulator of the tissue-specific glucocorticoid availability in cortisone reductase deficiency (OMIM: 604931) (Draper *et al.,* 2003). Indeed, in a study conducted on four patients suffering from cortisone reductase deficiency, four novel and one known mutations in the H6PDH gene in homozygous or compound heterozygous state were identified. Expression data on these mutations revealed loss of H6PDH function (Lavery *et al.,* 2008). Mouse models carrying the H6PDH mutations develop fasting hypoglycaemia, increased insulin sensitivity and increased basal and insulin-stimulated glucose uptake (Lavery *et al.,* 2008). It was observed that cortisol reductase deficiency presents a similar phenotype to polycystic ovary syndrome (POS). Furthermore, the H6PDH gene was associated with multiple sclerosis (Alcina *et al.,* 2010).

## VII. HOST–PATHOGEN INTERACTIONS: THE ROLE OF THE PPP IN INFECTIOUS DISEASE

### (1) The PPP as a target in parasitic protozoa

Protozoan parasites are responsible for a considerable number of debilitating infections that affect a significant number of people around the world, most commonly in developing countries. These protozoa include the kinetoplastids *Trypanosoma brucei*, *Trypanosoma cruzi* and *Leishmania* spp. These parasitic protozoa cause sleeping sickness, Chagas’ disease and leishmaniasis (cutaneous, visceral and mucosal), respectively. Various species of the Aconoidasida genus *Plasmodium* are responsible for malaria, while the archeamoeban (amitochondriate) parasite *Entamoeba histolytica* is the causative agent of amoebiasis, a disease characterized by diarrhoea (amoebic colitis) or abscesses principally of the liver. The biology behind the host–parasite relationship, the infection process and in some cases, even to identify drug targets, is highly dependent on parasite metabolism. Parasite metabolic reconfiguration and mutations in their enzymes may contribute towards resistance to drug treatment as well as parasite evasion of the host innate immune response. One important way the host immune system counteracts parasite infection is *via* the generation of hydrogen peroxide and other oxidants. Due to its function in maintaining the supply of the antioxidant cofactor NADPH, the PPP is therefore of great importance to the pathology of these parasites, becoming an attractive target for drug design.

#### (a) Kinetoplastids, the trypanothione pathway and the compartmentalization of the PPP in glycosomes

**Fig. 5:**
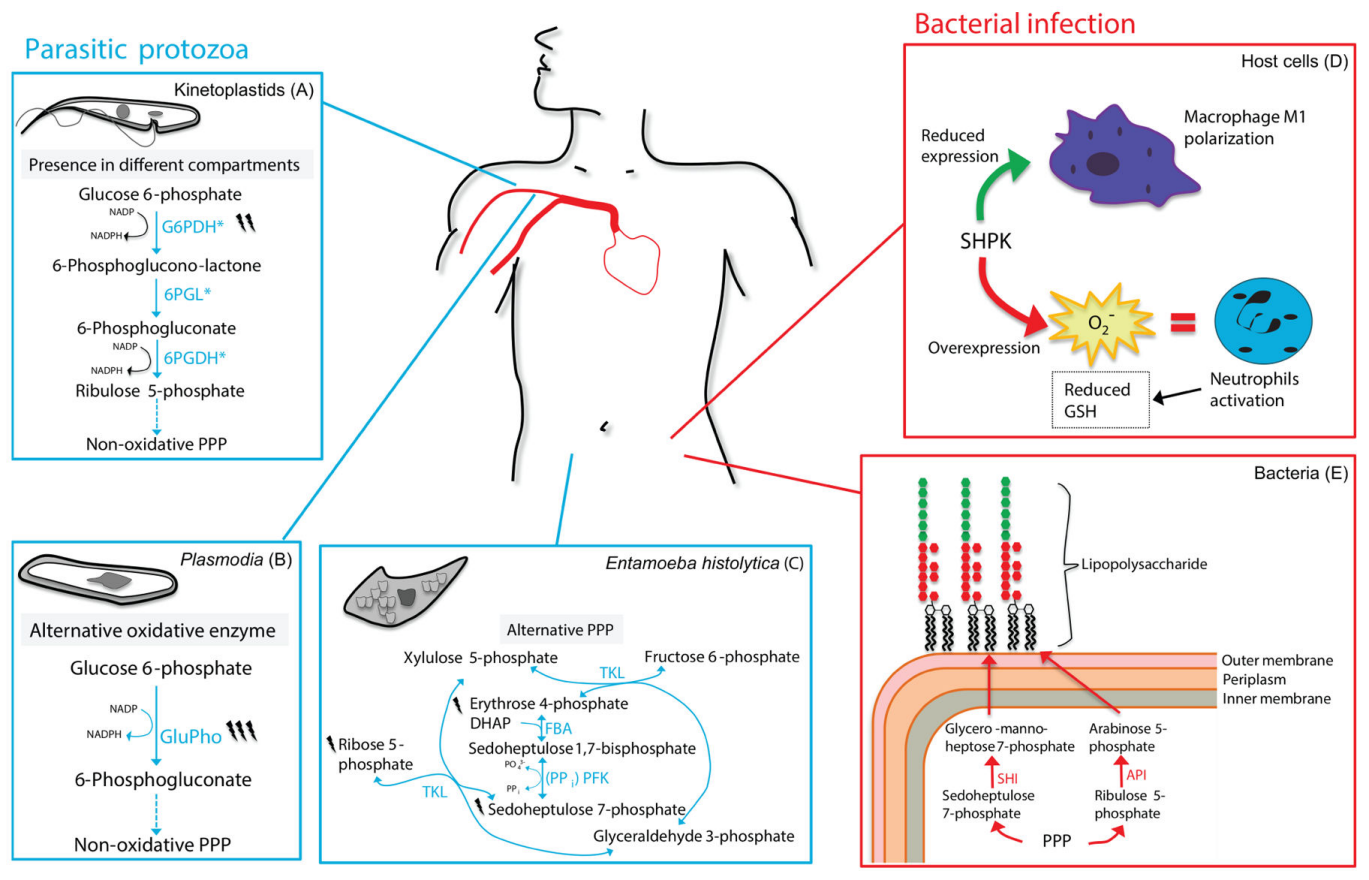
The pentose phosphate pathway (PPP) in parasitic protozoa (left) and bacterial infection (right). In kinetoplastids (A), PPP enzymes are localised in the cytosol and glycosomes (*). Plasmodia (B) have a bifunctional enzyme (glucose 6-phosphate dehydrogenase 6-phosphogluconolactonase; GluPho) that has the activity of glucose 6-phosphate dehydrogenase (G6PDH) and 6-phosphogluconolactonase (6PGL). *Entamoeba histolytica* lacks G6PDH and transaldolase (TAL), however has developed an alternative hexose–pentose interconversion pathway (C) in which the enzymes transketolase (TKL), fructose-bisphopshate aldolase (FBA) and pyrophosphate dependent-phosphofructokinase [(PPi)PFK] are involved. The activity of the PPP pathway is modulated by metabolites that respond to oxidants (i.e. H_2_O_2_ or paraquat); oxidant-responsive enzymes; or by the glutathione/glutathione disulfide (GSH/GSSG) ratio. 6PGDH, 6-phosphogluconate dehydrogenase. During bacterial lipopolysaccharide (LPS) infection of the mammalian intestine, sedoheptulokinase (SHPK) is of reduced activity in the host (D) and leads to macrophage M1 polarization. In bacteria (E), sedoheptulose 7-phosphate isomerase (SHI) and arabinose 5-phosphate isomerase (API) enzymes can increase LPS production. O_2_^−^: superoxide.

Kinetoplastids have a complete and functional PPP. Studies in both *T. cruzi* (Igoillo-Esteve *et al.,* 2007) and *Leishmania mexicana* (Maugeri *et al.,* 2003) have shown that the PPP metabolized 5–10% of total glucose. Most of the canonical PPP enzymes have homologues in these parasites and have been cloned, characterized and crystallized in at least one of the trypanosomatids (Fig. 5A) (Barrett *et al.,* 1994; Phillips *et al.,* 1998; Duffieux *et al.,* 2000; Veitch *et al.,* 2004; Igoillo-Esteve & Cazzulo, 2006; Stern *et al.,* 2007; Stoffel *et al.,* 2011; Kaur *et al.,* 2012). Exceptions include TAL and RPE, for which less information is available (Cronín, Nolan & Voorheis, 1989; Igoillo-Esteve *et al.,* 2007). However, these PPP enzymes could be important for the infection process. It has been observed that bloodstream forms (host stage) in *T. brucei* have neither TKL nor RPE activities; hence, at this stage, they are not capable of forming glyceraldehyde 3-phosphate from the non-oxidative branch of the PPP (Cronín *et al.,* 1989).

The enzymes of the PPP in these parasites are mainly cytosolic. However, they have also been allocated to the glycosome, the trypanosomatids’ peroxisome, that contains the major part of the glycolytic pathway (Hannaert *et al.,* 2003). The role of the PPP enzymes in the glycosome appears to be: (*i*) the exchange of intermediates with the glycolytic pathway; (*ii*) the supply of ribose 5-phosphate for nucleotide biosynthesis, which also occurs in the glycosome; and (*iii*) the supply of NADPH for the antioxidant system (trypanothione reductase) that has also been detected in the glycosome (Hannaert *et al.,* 2003). In turn, one of the main functions of the PPP in the cytosol is the supply of reducing power (NADPH) to the antioxidant system. In the trypanosome, antioxidant action relies on an alternative molecule, trypanothione [T(SH)_2_; N1-N8-bisglutathionylspermidine], an analogue of glutathione. Together with its reducing enzyme trypanothione reductase (TryR), trypanothione replaces all the functions that the system glutathione (GSH)/glutathione reductase (GR) has in other cells (Olin-Sandoval, Moreno-Sánchez & Saavedra, 2010). The major part of the antioxidant system in trypanosomatids therefore depends on trypanothione, and its reduction requires NADPH equivalents supplied by the PPP (Barrett, 1997). Due to the central role of the PPP in antioxidant metabolism, several studies have focused on the regulation of PPP enzymes under oxidative stress. It has been demonstrated that G6PDH increases its activity 46-fold in metacyclic trypomastigotes of *T. cruzi* (infective form) exposed to 70 μM H_2_O_2_, an increase related to an increment in protein content. By contrast, epimastigotes (insect form) exposed to 20 μM of H_2_O_2_ had decreased G6PDH activity and protein content, an observation which can be explained with the reasoning that under physiological conditions, epimastigotes are not exposed to oxidative stress (Igoillo-Esteve & Cazzulo, 2006). These results demonstrate that oxidative stress not only regulates the activity of G6PDH kinetically but also at the protein level.

RNAi-mediated knock-down of G6PDH in bloodstream forms of *T. brucei* promoted a negative effect on parasite growth, suggesting an essential function for this enzyme (Cordeiro, Thiemann & Michels, 2009). This PPP enzyme appears to be associated with the antioxidant machinery of the parasite *T. brucei* partially depleted of G6PDH are sensitive to H_2_O_2_ (Gupta *et al.,* 2011). Moreover, PPP flux in *Leishmania mexicana* increases when the parasites are exposed to methylene blue causing oxidative stress, providing further support that the PPP is highly essential in these parasites’ response to oxidative conditions (Maugeri *et al.,* 2003). The importance of the PPP in the response to oxidative stress in trypanosomes has also been corroborated by dynamic modelling. Recently, a kinetic model of glycolysis in *Trypanosoma brucei* (Albert *et al.,* 2005; Achcar *et al.,* 2012) was extended with PPP reactions (Kerkhoven *et al.,* 2013). Both models predicted that the flux through the cytosolic PPP was regulated by oxidative stress. Under low-oxidative-stress conditions the flux through this pathway was very low. However, the simulation of oxidative stress promoted an increase in the flux through glucose 6-phosphate dehydrogenase/hexose 6-phosphate dehydrogenase, 6-phosphogluconolactonase (PGL) and ribose 5-phosphate isomerase (RPI) of about seven-, 5.5- and 4-fold, respectively. After the stress, a steady state was predicted to be reached after 1 min (Kerkhoven *et al.,* 2013).

6PGDH in *T. brucei* is essential (Barrett, 1997; Kerkhoven *et al.,* 2013). The lethal phenotype has been attributed to the accumulation of 6-phosphogluconate inhibiting PGI (Barrett, 1997), a result that has been challenged by (*i*) the observation that fructose supplementation, which enters glycolysis after PGI, does not rescue the cells from death, and (*ii*) a lack of support from dynamic modelling (Kerkhoven *et al.,* 2013).

Mutations in TKL (null mutants), by contrast, do not have any effect on cell growth, nor were changes in morphology detected (Stoffel *et al.,* 2011). Metabolite analysis of these mutants showed that the substrates ribulose 5-phosphate, ribose 5-phosphate and xylulose 5-phosphate had accumulated 7.9-fold with a concomitant decline in the product sedoheptulose 7-phosphate. Additionally, intracellular concentration of 2,3-bisphosphoglycerate, phosphoenolpyruvate, fructose 6-phosphate and glyceraldehyde 3-phosphate were reduced 2-, 4.5-, 1.5- and 3.2-fold, respectively (Stoffel *et al.,* 2011). Consequently, taken together, these results indicate that a main role of the PPP in trypanosomatids appears to be in defence against oxidative stress.

#### (b) Plasmodium *spp*

*Plasmodium* spp. parasites possess a complete and functional PPP (Preuss, Jortzik & Becker, 2012). This has been confirmed through *in silico* analysis, using transcriptome profiles collected hourly during the intra-erythrocytic cycle of the parasite. Both branches of PPP are active at least at 50 hours post-invasion of the erythrocyte and their expression varies most likely due to cell requirements, such as ribonucleotide synthesis or NADPH and ATP demand (Bozdech & Ginsburg, 2005). The canonical PPP enzymes such as TKL and RPI have been cloned and characterized in *P. falciparum* (Holmes *et al.,* 2006; Joshi *et al.,* 2008). Interestingly, *Plasmodium* spp. possess fusion enzymes within the PPP. One well-studied example is a protein that combines G6PDH and 6PGL activities in a single enzyme, glucose 6-phosphate dehydrogenase 6-phosphogluconolactonase (GluPho) (Fig. 5B). This enzyme is regulated by S-gluthathionylation, suggesting that the glutathione/glutathione disulfide (GSH/GSSG) ratio regulates GluPho activity (Jortzik *et al.,* 2011). The fusion protein is not the exception, and multiple independent fusions of G6PDH with other PPP enzymes have been found in this plasmodial parasite, indicating that pathway efficiency is potentially increased by channelling metabolites in this manner (Stover, Dixon & Cavalcanti, 2011).

Plasmodial parasites are continuously exposed to oxidative stress during the life-cycle stage in the erythrocyte; as they take haemoglobin into their acid food vacuole, Fe^2+^ is oxidised to Fe^3+^ and superoxide anions are produced, which in turn promote the formation of H_2_O_2_ and hydroxyl radicals. The reductases involved in these parasite’s antioxidant systems are NADPH-dependent glutathione and thioredoxin reductases (Müller, 2004). Although the parasites have the enzymes isocitrate dehydrogenase and glutamate dehydrogenase, which are also able of supplying NADPH to the cell, the former enzyme generally oxidizes this cofactor and the latter has been demonstrated not to be an NADPH supplier for antioxidant systems (Preuss *et al.,* 2012). Furthermore, ribose 5-phosphate can be obtained from the uptake and degradation of host purines in contrast to being obtained from the PPP. Thus, the main role of the PPP in *Plasmodium* spp. seems to be to supply NADPH.

The role of the PPP in *P. falciparum* is indispensable during parasite infection. The parasite’s pathway contributes to 82% of total PPP activity in infected red blood cells (IRBCs) and 72% in G6PDH- deficient IRBCs (Atamna, Pascarmona & Ginsburg, 1994). The highest NADPH demand appears when the parasite has reached maturation (trophozoite stage) in IRBCs, which is consistent with an increase in G6PDH activity in IRBCs during this period (Atamna *et al.,* 1994). As discussed in Section VI.1, G6PDH- deficient hosts (46% females heterozygous; 58% males hemizygous) have increased resistance to malaria (Cappellini & Fiorelli, 2008). Although this resistance is not fully understood, two hypotheses have been proposed: one suggests that the oxidative stress in G6PDH- deficient erythrocytes may be the cause of impaired infection; the other proposes that these infected erythrocytes are more easily recognized and destroyed by the host’s immune system (Müller, 2004).

#### (c) The alternative PPP of Entamoeba histolytica

An alternative PPP is found in the parasite, *Entamoeba histolytica*. This parasite lacks G6PDH, 6PGDH and TAL (Barrett, 1997; Husain *et al.,* 2012) and has developed an alternative hexose–pentose interconversion pathway for the formation of pentose phosphates. This alternative pathway is constituted of several reactions catalysed by TKL, FBA and phosphofructokinase (PPi-dependent) (Susskind, Warren & Reeves, 1982) (Fig. 5C). When *Entamoeba histolytica* is exposed to oxidative stress, metabolites of the non-oxidative branch of the PPP, glycerol and chitin biosynthesis are increased, a process attributable to an inhibition of glycolytic enzymes which, in turn, promotes redirection of the carbon flux (Husain *et al.,* 2012). Thus, although the oxidative branch is absent from the PPP in this parasite, it seems that the non-oxidative pathway still responds to the presence of oxidative stress. This observation is analogous to budding yeast, where the non-oxidative PPP plays an NADPH-independent role in the stress response too (Krüger *et al.,* 2011).

### (2) The PPP in bacterial infection

Similar to eukaryotes, the PPP and glycolysis (together with overlapping reaction sequences such as the Entner–Doudoroff pathway) constitute core carbon metabolism in bacteria (Sprenger, 1995). As in eukaryotes, the PPP is required to provide NADPH equivalents, nucleotides and sugar phosphate precursors. An important additional function however concerns the provision of sedoheptulose 7-phosphate for the initiation of lipopolysaccharide (LPS) biosynthesis (Taylor *et al.,* 2008). Moreover, the PPP appears to be the only pathway allowing bacteria to utilize sugars such as 3-xylose, 3-ribose, and 3-arabinose, which cannot be catabolised by other means (Wood, 1985; Sprenger, 1995; Lin, 1996). Here we briefly introduce the role of the PPP in the bacterial infection process and the importance of this pathway for both host and pathogen.

#### (a) The PPP is required in the host response to fight microbial infection

The invasion of host cells gives rise to the activation of defence mechanisms required for survival and pathogen expulsion. The host response is characterized by microbial sensors activating signalling pathways and inducing effector mechanisms (Nish & Medzhitov, 2011). Macrophages are responsible for the host immune response by inducing signalling modules and alterations in cell morphology and metabolic function (Martinon, Mayor & Tschopp, 2009; Haschemi *et al.,* 2012). The cellular reprogramming utilizes metabolic adaptation in response to environmental changes. Nevertheless, there is only limited knowledge of the bioenergetic rearrangement that takes place during macrophage activation. Recently SHPK [formerly carbohydrate kinase- like (CARKL)] has been identified as a modulator of macrophage regulation during LPS-induced infection (Haschemi *et al.,* 2012). Reduced SHPK expression was observed in both *in vivo* and *in vitro* experiments and associated with macrophage type 1 (M1) polarization. By contrast, SHPK overexpression induced a pro-inflammatory response, characterized by the presence of nuclear factor kappa-B (NF-*κ*B), and an increase in the production of intracellular superoxide radicals (probably due to sustained sedoheptulose 7-phosphate formation), which is similar to the neutrophil-induced response (Haschemi *et al.,* 2012) (Fig. 5D).

Neutrophil activation characterizes the immune response in the Gram-negative bacterium *Helicobacter pylori*, the agent causing chronic gastritis and peptic ulcer disease (Nielsen *et al.,* 1994; Basso, Plebani & Kusters, 2010). *H. pylori* was the first microorganism to be directly associated with stomach carcinogenesis (Sipponen & Hyvärinen, 1993). It was suggested that, following *H. pylori* infection, neutrophil activation rapidly increases ROS production (Obst *et al.,* 2000). Moreover, GSH availability, normally high in the stomach, is rapidly depleted after *H. pylori* infection of the human gastric mucosa, mainly because GSH is used for repairing oxidative DNA lesions in gastric carcinogenesis (Shirin *et al.,* 2001; Basso *et al.,* 2010). By contrast, GSH levels were increased in mouse model infections, leading to the hypothesis that the GSH (and the oxidative PPP, regulating GSH synthesis) could be directly involved in the response to the oxidative stress induced by *H. pylori* infection (Matthews & Butler, 2005).

#### (b) The PPP is a central pathway for bacterial infection and LPS biosynthesis

The infection process requires rapid adaptation of intracellular and extracellular bacteria, involving reconfiguration of their central carbon metabolism. This is caused predominantly by their newly encountered physical conditions and nutrient availability (Eisenreich *et al.,* 2010; Swanepoel & Loots, 2014). More precisely, pathogens need to modulate their metabolism and coordinate their life cycle in order to develop specific virulence factors (Ray *et al.,* 2009; Eisenreich *et al.,* 2010).

Due to the presence of different microenvironments (or niches), the host environment is characterized by the availability of several nutrient sources (Brown, Palmer & Whiteley, 2008). For example, enterohaemorrhagic (EHEC) and uropathogenic (UPEC) *Escherichia coli* strains (causing haemolytic colitis and urinary tract infection, respectively), differ in their ability to cause infection according to their localization (i.e. nutrient availability) (Alteri & Mobley, 2012). The mammalian urinary tract is characterized by the presence of amino acids and small peptides, therefore mutations in the genes coding for the PPP and glycolytic enzymes do not affect the pathogenicity of the UPEC *E. coli*. On the other hand, EHEC *E. coli* needs to up-regulate both glycolysis and PPP in order to colonize the host intestine, due to the high levels of glycogen available which can be used as an external carbon source (Alteri & Mobley, 2012).

Last but not least, the PPP has been found to play an essential role in the biosynthesis of lipopolysaccharides (LPSs). LPS is part of the external layer of Gram-negative bacteria and is involved in not only bacterial protection but also in the activation of the host immune response (Raetz & Whitfield, 2002). The biosynthesis of LPS has been intensively studied in order to provide new therapeutic agents against Gram-negative pathogens (Wang & Quinn, 2010). One of the possible targets for drug discovery is SHI, as characterized in *E. coli, Pseudomonas aeruginosa* (Taylor *et al.,* 2008) and *H. pylori* (Sarkar *et al.,* 2012). This enzyme converts sedoheptulose 7-phosphate from the PPP into the lipopolysaccharide precursor glycero-manno-heptose 7-phosphate (Kneidinger *et al.,* 2001; Valvano *et al.,* 2002; Taylor *et al.,* 2008), and has therefore become a central target for the development of new antibiotics and adjuvants. Further investigations on the LPS biosynthetic pathway highlighted another enzyme involved in the pathogenicity of an *E. coli* UPEC strain, arabinose 5-phosphate isomerase (API), which converts the PPP sugar ribulose 5-phosphate into the LPS precursor arabinose 5-phosphate (Mosberg *et al.,* 2011) (Fig. 5E), emphasizing these PPP enzymes as attractive targets for the development of future antibiotics.

## VIII. THE ROLE OF THE PPP IN CELL PROLIFERATION AND STEM CELLS

Cell growth necessitates biosynthesis of the required intermediates, such as nucleotides, amino acids, and lipid precursors. Consequently, when proliferation is induced, cells restructure their central carbon metabolism in order to adapt to the rise in metabolic demands. This metabolic reconfiguration includes the shuffling of the energy flux outside the mitochondria to fuel glycolysis and the PPP (Levine & Puzio-Kuter, 2010; Vander Heiden *et al.,* 2010; Grüning & Ralser, 2011; Yamamoto *et al.,* 2014). The PPP playing a crucial, non-redundant role in the supply of building blocks such as ribose 5-phosphate, the molecular backbone of nucleic acids, is consequently of central importance. Indeed, a key feature of the metabolic transformation events accompanying cellular proliferation is the enhancement of biosynthetic capacity. Hence, diverting the energy flux towards the non-oxidative branch of the PPP has the key advantage of enabling the needed nucleotide biosynthesis through the production of ribose 5-phosphate (Deberardinis *et al.,* 2008). In addition, this metabolic restructuring safeguards the cellular redox balance, by modulating NADPH production in the PPP.

Human pluripotent stem cells (PSCs), including embryonic stem cells (ESCs) (Thomson *et al.,* 1998) and induced pluripotent stem cells (iPSCs) (Takahashi *et al.,* 2007), are of particular interest in biomedicine given their ability to proliferate indefinitely (self-renewal) and to differentiate into virtually any cell type of the body (pluripotency). The central importance of metabolism in reprogramming and self-renewal has recently caught the attention of the stem cell community, with most discoveries dating back to recent years only (reviewed in (Folmes *et al.,* 2012; Zhang *et al.,* 2012; Xu *et al.,* 2013; Bukowiecki, Adjaye & Prigione, 2014) and has revealed that the induction and maintenance of PSCs is associated with a profound change of anabolic demands. PSCs exhibit an elevated rate of proliferation and distinct cell cycle features compared to common somatic cells (Ruiz *et al.,* 2011). Moreover, PSCs are particularly sensitive to redox imbalance (Saretzki *et al.,* 2008) and display low levels of oxidatively modified proteins, lipids, and DNA (Prigione *et al.,* 2010). Indeed, increased ROS levels have been shown to promote differentiation (Yanes *et al.,* 2010).

The establishment of the proliferative PSC state has been found to be coupled with elevated lactate generation and enhanced glycolytic flux (Prigione *et al.,* 2010; Folmes *et al.,* 2011). Moreover, upon glycolysis-activating conditions, such as under hypoxia stimulation or after treatment with small-molecule inducers of the master metabolic regulator hypoxia-inducible transcription factor 1 (HIF-1) (see section VIII-4-d), the efficiency of somatic cell reprogramming is significantly improved (Yoshida *et al.,* 2009; Zhu *et al.,* 2010). Conversely, genetic ablation of HIF-1 hampers the formation of iPSCs (Prigione *et al.,* 2013; Mathieu *et al.,* 2014). Finally, increased expression of the pyruvate kinase isozyme M2 (PKM2), which regulates the flux distribution between glycolysis and the PPP (section VIII-3), has been identified in both ESCs and iPSCs compared to somatic cells (Prigione *et al.,* 2013).

Currently, evidence from altered PPP metabolism in stem cell populations mainly originates from the analysis of their gene expression profiles. Genes regulating the first steps of glycolysis were up-regulated in PSCs compared to somatic cells, including glucose uptake (*SLC2A3*) and glucose phosphorylation to glucose 6-phosphate (*HK3* and *GCK*) (Prigione *et al.,* 2011; Varum *et al.,* 2011). Enzymes of the final steps of glycolysis (such as *PGAM2, ENO, PKLR*, and *LDH*) are up-regulated in PSCs. On the other hand, the expression level of the glycolytic enzymes downstream of glucose 6-phosphate, including *GPI, PFK,* and *ALDO*, is reduced in PSCs, while the level of genes involved in the non-oxidative branch of the PPP (*RPIA* and *TKT*) is augmented (Folmes *et al.,* 2011; Prigione *et al.,* 2011; Varum *et al.,* 2011). Hence, the transcriptional data of PSCs suggest that glycolytic intermediates may be diverted into the PPP, in order to support both the biomass accumulation and redox homeostasis that are associated with the maintenance and derivation of PSCs. Accordingly, LC-MS/MS-based metabolite quantification detected the accumulation of glucose 6-phosphate and decreased dihydroxyacetone phosphate in PSCs compared to fibroblasts (Prigione *et al.,* 2011), which may be indicative of overall increased metabolic activity due to proliferation and possibly specific PPP activation. Elevated protein expression of hexokinase II (HXK2), which causes a higher glycolytic rate than isoform I, has also been observed in the mitochondria of PSCs (Varum *et al.,* 2011). This is of particular interest since HXK2 activity may be stimulated under hypoxia by the p53-inducible target TP53-induced glycolysis and apoptosis regulator (TIGAR) (see sectionVIII-4-a) to induce the PPP and preserve redox homeostasis (Cheung, Ludwig & Vousden, 2012).

Finally, the importance of the PPP for the maintenance of the pluripotent state is supported by the findings that G6PDH-depleted ESCs proliferate at a reduced rate and, upon oxidant exposure, are incapable of increasing the PPP flux, thus resulting in apoptotic cell death (Filosa *et al.,* 2003; Fico *et al.,* 2004). Furthermore, genetic or small-molecule-based inhibition of the PPP forces PSCs to exit the self-renewal state and start the differentiation process (Manganelli *et al.,* 2012). Overall, it appears that promoting the activation of the PPP is functionally critical to support the establishment and the maintenance of the proliferative conditions associated with the undifferentiated PSC state. It is however not clear to which extent the metabolic reconfiguration has an active role in maintaining pluripotency, or whether changes in energy metabolism are causative in driving differentiation. Evidence for an active role of the PPP in supporting cell proliferation has however been found by studying the metabolism of cancer cells.

### (1) The role of the PPP in deregulated cell proliferation and the pathogenesis of cancer: the Warburg effect

Rising attention has recently been paid to deregulated cell proliferation when it has been noticed that malignant transformation and metabolic reprogramming may be intimately intertwined. Despite a vast amount of research, cancer still represents the second most common cause of death in the world, beaten to the top only by cardiovascular diseases. While the last decade has substantially changed the way cancer therapy is performed, the majority of newly approved molecular-targeted drugs (e.g. antibodies against growth factors or their receptors, tyrosine kinase inhibitors and other small molecules) failed to result in significant and long-lasting improvement of therapy efficacy (Fojo & Parkinson, 2010). This is partially explained by the hallmark genomic instability of malignant cells that results in an impressive propensity to adapt to and, ultimately, resist inactivation of ‘cancer-specific’ signalling pathways (Bock & Lengauer, 2008; Gillies, Verduzco & Gatenby, 2012). Inhibition of processes that are absolutely essential and non-redundant for tumour cell proliferation is a promising strategy to improve cancer therapy. Tumour-specific metabolism clearly represents such a process. Despite being recognized nearly a century ago, the fundamental importance of metabolic deregulation for cancer pathogenesis has escaped the appreciation of most cancer researchers for decades. It was not until the post-genome era that metabolic reprogramming was widely accepted as an emerging hallmark of cancer (Hanahan & Weinberg, 2000; Hanahan & Weinberg, 2011). Recently however, characterization of cancer metabolism has become the focus of a rapidly growing research community, taking advantage of the improved analytical and increasingly also computational methodology to identify fascinating and unexpected interactions (Weinberg, 2014). Observed changes in metabolism are in no way ‘trivial’, indeed can be quite specific dependent on both the responsible genetic lesion and tumour tissue type (Yuneva *et al.,* 2012). While the majority of published work analysed the role of glycolysis, glutaminolysis and mitochondrial activity, the importance of the PPP for malignant transformation remained elusive for quite some time.

Otto Warburg was not only able to identify some of the first enzymes and co-enzymes of central metabolism, but at the same time, was one of the first research pioneers that recognized the importance of altered metabolism for tumour growth. He and his co-workers discovered an increase in glucose uptake and lactate production in concert with a decrease in oxygen uptake (known as the ‘Warburg effect’), reviewed in (Warburg, 1956). Intriguingly, the elevation in glycolytic flux also occurred under sufficient oxygen supply (aerobic glycolysis). Based on these results, Warburg concluded that cancer cells must suffer from defects in their respiratory machinery. Today, we know that although many cancers show reduced activity of oxidative phosphorylation (OXPHOS; (Ferreira, 2010; Cairns *et al.,* 2011)), most cancers [with important exceptions, oncocytoma for instance (Mayr *et al.,* 2008)], possess a fully functional respiratory chain and are therefore biochemically fully capable of using the respiratory chain for ATP production.

But why are cancer cells then not fully exploiting this efficient ATP-producing machinery? A cutback in OXPHOS seems counterintuitive since rapid proliferation demands large amounts of energy. This observation implies that other factors than ATP production are more limiting for the cancer cell. Recent observations indicate that a balanced redox state, achieved in part by increased PPP activity, is essential for tumourigenesis (Anastasiou *et al.,* 2011; Grüning & Ralser, 2011; Tosato *et al.,* 2012). A more disputed theory concerns the cross-feeding of cancer cells through lactate, termed the ‘Reverse Warburg effect’ (Pavlides *et al.,* 2009). Although this theory has attractive components, it fails to explain some aspects of the effect; for instance it does not explain why other organisms, like yeast cells, also show Warburg-like metabolic reconfigurations despite not sharing lactate. Importantly, however, all these studies indicate that the often heard hand-waving explanation ‘Respiration is reduced in cancer cells to save carbon equivalents for biosynthesis’ is to be questioned as a cause for the Warburg effect: First, respiratory activity does not compete with aerobic glycolysis for carbons, as aerobic glycolysis is followed by lactate excretion instead of pyruvate decarboxylation. Second, also the PPP contains a CO_2_-producing reaction. Thus, Warburg like cells have a negative carbon balance.

Oxidative stress is a major cause of damage for macromolecules and can eventually lead to cell death. On the other hand, a certain amount of oxidizing equivalents are certainly necessary for cell physiology, and thus, only situations with constantly or periodically elevated ROS levels can be considered as a risk factor for tumourigenesis. ROS-induced DNA damage can lead to cancerogenic mutations and genomic instability, and ROS also trigger inflammatory pathways and have a stabilizing effect on HIF-1; a transcription factor highly expressed in cancer cells (Gao *et al.,* 2007; Perera & Bardeesy, 2011; Wu & Le, 2013). Therefore, the pool of intracellular ROS must be kept balanced and below a toxic threshold – a drastic shift towards oxidation would cause tumour cell death (Gao *et al.,* 2007; Perera & Bardeesy, 2011). Thus, dynamic tuning of the metabolic network as well as the antioxidant systems, involving glycolytic metabolite accumulation and PPP activation, is of fundamental importance to keep the production of cellular building blocks, energy and reducing equivalents in check. *Vice versa* however, pro-oxidant therapies could prove helpful in inhibiting tumour cell proliferation.

### (2) Evidence for enhanced PPP activity in cancer cells

Gene and protein expression analyses together with immunohistochemistry are widely applied as surrogate methods to assess the role of specific factors for cancer pathogenesis. However, while these methods are certainly useful and have helped to identify numerous molecules important for cancer biology, a valid and detailed characterization of metabolic pathways cannot be achieved by them. Metabolic pathways appear mostly regulated by post-translational mechanisms (Daran-Lapujade *et al.,* 2004; Buescher *et al.*, 2012; Kochanowski, Sauer & Chubukov, 2013). In addition, flux of the PPP is dependent on the level of co-factors (NADP^+^ for the oxidative PPP), substrate availability (non-oxidative PPP), and the flux through glycolytic enzymes. Hence, information about the abundance of mRNA and also protein levels is limited to pinpoint accurately changes in PPP activity and their potential causal importance for cancer biology, so it is required to determine these values in concert with flux and/or metabolite concentrations. Due to the difficulty in applying these techniques in heterogeneous tumours, it is hence not surprising that published literature is rather scant. Fortunately, G6PDH is an informative exception as its enzyme activity in tumours is well studied and was increased in various human cancer types when compared to the respective benign control tissue, e.g. cervix, uteri, prostate and breast (Pedersen, 1975; Zampella, Bradley & Pretlow, 1982; Bezwoda *et al.,* 1985). To the best of our knowledge, there is less information available about tumour-specific activities of the non-oxidative PPP, especially for its key enzymes TAL and TKL.

Assessment of enzyme activity however points towards modified PPP flux in cancer. Stable isotope resolved metabolomics (SIRM), indicates enhanced PPP activity in the human breast cancer cell line MCF7 when compared to non-transformed mammary epithelial cells (Meadows *et al.,* 2008). Similar results have been obtained in renal cell carcinoma where altered activity of the PPP has been identified as a key metabolic feature of the cancer state (Catchpole *et al.,* 2011). In addition, PPP adaptation could be crucial for cancer cells that use glucose alternatives, such as fructose, for their carbohydrate needs. There is evidence that in pancreatic cancer cells, fructose is preferentially metabolized *via* the non-oxidative PPP supporting tumour growth (Liu *et al.,* 2010). In this context, a homologue of TKL, TKL-like protein (TKTL1) is detected in tumour tissue and its expression level has been correlated with the progression of cancer (Diaz-Moralli *et al.,* 2011; Kayser *et al.,* 2011). However, due to the difficulty in detecting TKTL1 enzymatic activity it is currently undecided whether TKTL1 participates in the PPP or not (Meshalkina *et al.,* 2013).

As an alternative to the classical biochemical approaches, functional imaging is becoming increasingly sophisticated and has shown promise as another more direct method to assess metabolic changes *in vivo*. A recent study used intravenous infusion of [1,2-^13^C_2_] glucose, followed by ^13^C NMR analysis of the micro-dissected tumour mass and non tumour-bearing surrounding brain, to assess PPP flux relative to glycolytic flux. The malignant tissue in this study did not show enhanced PPP flux relative to glycolysis, when compared with the surrounding benign brain tissue (Marin-Valencia *et al.,* 2012*a*; Marin-Valencia *et al.,* 2012*b*), and it was suggested that damage to the surrounding brain was confounding the measurements. While the latter two studies demonstrate the potential of measurements for the PPP flux, it is clear that additional data are needed from a broad spectrum of tumours for a more comprehensive picture of PPP activity in cancer.

New opportunities arise from the use of hyperpolarized NMR tracers, as these allow non-invasive and real-time assessment of metabolic flux *in vivo*. The technique has recently been translated to the clinic with a study in prostate cancer (Nelson *et al.*, 2013). Intriguingly, in studies on *E. coli*, yeast and cancer cells, *in vitro* signals from glycolytic intermediates have been detected, such as dihydroxyacetone phosphate, and also a signal that has been assigned to 6-phosphogluconate, offering the potential for *in vivo* measurements of PPP flux as well. Such measurements have recently been reported for tumors *in vivo*, where hyperpolarized [U-^2^H, U-^13^C] glucose and the lactate formed from it were imaged and the resonance previously assigned to 6-phosphogluconate was observed (Rodrigues *et al.,* 2014).

### (3) Enzymatic switches enable metabolic adaptation of cancer cells

Different enzymatic switches could be involved in triggering and modulating metabolic reprogramming towards increased PPP flux. Proliferating mammalian cells have increased levels of glycolytic enzymes, one of these is represented by PK isozymes M1/M2 (PKM1/2) (Bock & Lengauer, 2008; Christofk *et al.,* 2008; Hitosugi *et al.,* 2009; Bluemlein *et al.,* 2011). PK catalyses the ‘final’ step in glycolysis and converts PEP into pyruvate; a reaction which yields ATP, and is thus required for the net gain in glycolytic energy production (Fraenkel, 1986).

Most human tissues are dominated by the expression of one of two mutually exclusive spliceforms of the PKM gene: PKM1 and PKM2 (Mazurek *et al.,* 2005; Bluemlein *et al.,* 2011). In most human tissues whether healthy or cancerous, (bladder, liver, colon, lung, kidney, thyroid, fibroblasts, epithelial cells), but not in muscle and potentially neurons, PKM2 is the dominantly expressed isoform over PKM1 (Bluemlein *et al.,* 2011). At least liver and red blood cells express an additional PK isoform, known as PKLR (Zanella & Bianchi, 2000).

Among PKM-expressing tissues, the total PKM expression level and the distribution between the two isoforms varies significantly, and ranges from 55% PKM2 over PKM1 of total PKM in (muscle-rich) bladder tissue, to up to 96% PKM2 of total PKM in colon tissue. At the same time, in absolute values, the PKM content ranged from ~10 fmol/μg total protein in thyroid to 300 fmol/μg total protein in colorectal carcinoma (Bluemlein *et al.,* 2011). Hence, the expression level of the PKM gene appears to be an important determinant of total pyruvate kinase activity and is highly dependent on the tissue. In many cancer cells PKM2 expression is increased when compared to tissue-matched controls (Ashrafian *et al.*, 2010; Bluemlein *et al.,* 2011; Zhou *et al.,* 2012). Despite the total protein amount being up-regulated, overall PK activity however does not increase accordingly, or it is inhibited, suggesting that the specific PKM activity is lowered in the tumour cells (Hitosugi *et al.,* 2009; Vander Heiden *et al.,* 2010). This modulation of PK activity creates the opportunity to switch between high glycolytic flux, or induce a metabolic reconfiguration towards OXPHOS and an activated PPP. In contrast to PKM1 that is a tetramer with high constitutive activity (Imamura & Tanaka, 1972; Wu & Le, 2013), PKM2 can be flexibly tuned and allosterically modulated in activity and switches between monomeric, dimeric and tetrameric states (Morgan *et al.,* 2013). Allosteric activation of PKM2 can be achieved by interaction with fructose 1,6-bisphosphate, succinylaminoimidazolecarboxamide ribose 5′-phosphate (SAICAR) and serine (Chaneton *et al.,* 2012; Keller, Tan & Lee, 2012). In addition, post-translational modifications can modulate PKM2 activity. For example, phosphorylation at tyrosine 105 prevents the formation of the more active tetrameric form of PKM2 (Hitosugi *et al.,* 2009), and P300/CBP-associated factor (PCAF)-mediated acetylation on lysine 305 reduces activity (Wang *et al.,* 2010). During high ROS levels, lower PK activity could be essential to maintain cell survival. Similarly to respiring yeast (section III), cancer cells accumulate PEP because of reduced PK activity (Anastasiou *et al.,* 2011; Grüning *et al.,* 2011). Oxidation of PKM2 at cysteine 358 can decrease its activity and redirect glycolytic intermediates towards the PPP for the production of NADPH. Disruption of this mechanism can exacerbate oxidative stress and subsequently decrease proliferation in cancer cells (Anastasiou *et al.,* 2011).

What does link decreased PK activity and increased cellular oxidative capacity? The mechanism seems to depend on the glycolytic block and PEP accumulation. PEP interferes with other glycolytic reactions, such as phosphoglycerate mutase, glucokinase, GPI, PFK, FBA and TPI (Ogawa *et al.,* 2007; Fenton & Reinhart, 2009; Grüning *et al.,* 2011; Grüning *et al.,* 2014). The latter can directly lead to increased PPP activity and stress protection. Despite *in vitro* kinetics indicating a low flux control coefficient of TPI (Knowles & John, 1977), *in vivo* experiments detect increased PPP activity when TPI activity is only slightly compromised (Ralser *et al.,* 2007). TPI inhibition is sufficient to cause a block in upper glycolysis and an increase in PPP metabolites (Grüning *et al.,* 2011; Grüning *et al.,* 2014). This redirection of metabolites by the PK-PEP-TPI feedback loop enables cells to adapt to a higher level of ROS and protect from oxidative damage (Ralser *et al.,* 2007; Grüning *et al.,* 2014). TPI thus might represent a key enzymatic switch for metabolic reprogramming. Although low PK and TPI activity limit the ATP yield from energy metabolism, the cell’s redox balance is maintained which could be more important for cancer survival (Cairns *et al.,* 2011; Grüning & Ralser, 2011).

### (4) Interaction of the PPP with oncogenic pathways

#### (a) p53

**Fig. 6:**
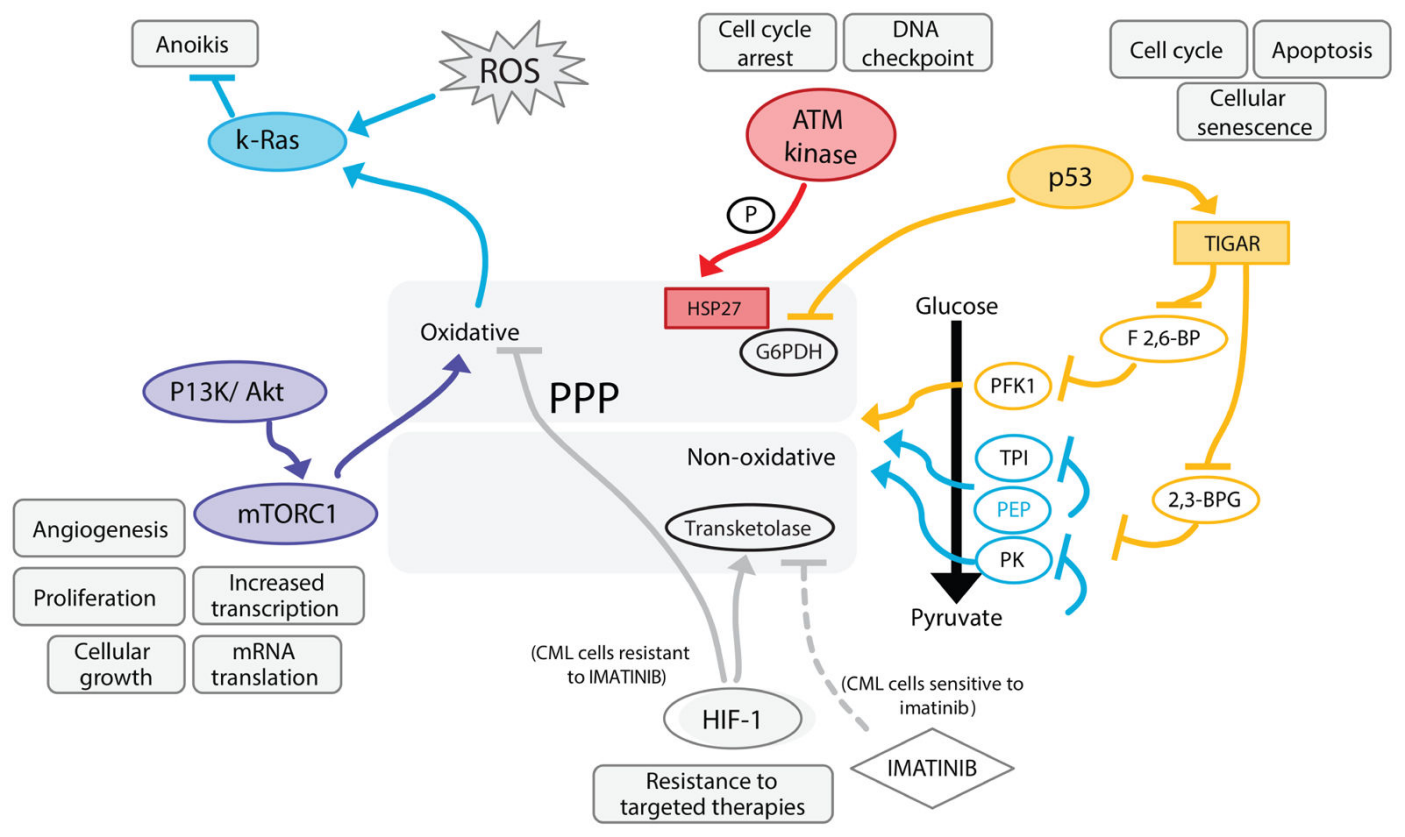
The pentose phosphate pathway (PPP) is associated with several cancer- and cell-proliferation-related signalling cascades. The p53 pathway can stimulate the PPP by inhibiting phosphofructokinase 1 (PFK1) through TP53-induced glycolysis and apoptosis regulator (TIGAR), and by inhibiting glucose 6-phosphate dehydrogenase (G6PDH). This enzyme is also targeted by the ataxia telangiectasia mutated (ATM) kinase, which increases G6PDH activity by phosphorylating heat shock protein 27 (HSP27), and an NAD-dependent deacetylase (SirT2), which acts on this enzyme directly by de-acetylation. All three mechanisms activate the oxidative branch of the PPP, which is also controlled by the mammalian target of rapamycin complex 1 (mTOR) pathway. Cancer signalling mechanisms operate alongside allosteric and metabolic regulation. For instance, reduced pyruvate kinase PKM2 activity leading to triosephosphate isomerase (TPI) inhibition increases the carbohydrate flux towards the PPP, achieving a metabolic self-regulation that counteracts oxidative stress. The activity of the PPP itself has an influence on cancer signalling pathways. The antioxidant capacity of the PPP modulates proto-oncogene *k-ras*-driven tumourigenesis; concurrently, reactive oxygen species (ROS) can potentiate the oncogenic activity of *k-ras* (the two opposing regulations are highlighted). The PPP has also been associated with drug resistance and hypoxia. Depending on the sensitivity to imatinib, chronic myeloid leukaemia (CML) cells can either exhibit reduced (sensitive cells) or increased (resistant cells) transketolase (TKL) activity after hypoxia-inducible transcription factor 1 (HIF-1) activation. F 2,6-BP, fructose 2,6-biphosphatase; P13K (Phosphatidylinositol-3-kinase), Akt (protein kinase B); PEP, phosphoenolpyruvate.

The transcription factor p53 represents a tumour suppressor with well-established functions on genomic integrity, apoptosis and cell cycle control (Vazquez *et al.,* 2008). p53 is the most commonly mutated gene in human cancers and its loss constitutes a pivotal mechanism of therapy failure (Bensaad *et al.,* 2006; Rohwer *et al.,* 2010). It became evident in recent years that p53, in addition to the functions outlined above, exerts control over metabolic pathways. The p53 target gene TIGAR was shown to dampen glycolysis by lowering the level of fructose 2,6-bisphosphate which is a powerful allosteric activator of PFK1. As a result the glycolytic intermediates can be diverted to the oxidative or non-oxidative branch of the PPP (Fig. 6). This leads to decreased cellular levels of ROS due to the action of NADPH, ultimately resulting in enhanced cell survival and growth (Bensaad *et al.,* 2006). In highly proliferative tissues such as the intestine, a lack of TIGAR *in vivo* leads to decreased regeneration after acute stresses such as ulcerative colitis and irradiation, indicating an important role of TIGAR in proliferation (Cheung *et al.,* 2013). Consistently, overexpression of TIGAR has been observed in a number of tumour types, and also in invasive cancer cells compared to normal tissues (Wanka, Steinbach & Rieger, 2012; Won *et al.,* 2012; Cheung *et al.,* 2013). In an *in vivo* model of intestinal adenoma where adenomatous polyposis coli (APC) is deleted in LGR5+ intestinal stem cells, TIGAR deficiency decreases tumour burden and average tumour size, which results in increased disease-free survival in these mice. Tumour intestinal crypts isolated from these mice showed that the *in vitro* growth of the TIGAR- deficient tissues can be rescued by the addition of antioxidants and nucleosides, again indicating an important role of TIGAR in increasing PPP during proliferation. While the role of TIGAR in promoting tumour growth seems to be counterintuitive to p53 as a tumour suppressor, TIGAR expression is uncoupled from p53 expression in various cell lines (Cheung *et al.,* 2013). Hence, it is possible that a p53 target protein such as TIGAR can become oncogenic when it is not properly regulated by p53. Recently, it has been shown that TIGAR predominantly functions as phosphoglycolate-independent 2,3-bisphos phoglycerate phosphatase (Gerin *et al.,* 2014). Another interpretation is thus that p53 could stimulate the non-oxidative PPP *via* TIGAR by directly targeting lower glycolysis. In contrast to this activity of a p53-target gene, p53 itself can inhibit G6PDH and regulate its activity; this results in reduced PPP activity and a redirection of the central carbon flux towards increased glycolytic activity (Jiang *et al.,* 2011). As a result, p53-deficient cells display enhanced lipid synthesis as well as reduced sensitivity towards oxidative stress-induced cell death as a functional consequence of higher oxidative PPP activity (Jiang *et al.,* 2011). Taken together, p53 seems to influence the PPP antithetically, both as an inhibitor (*via* direct influence on G6PDH) and as an activator (*via* TIGAR). These opposing functions of p53 may reflect the different roles of p53 depending on the severity of the damage to the cell. During transient and mild stress, p53 may act as a pro-survival mediator for repair and regeneration. However, if the damage is too high and persistent, p53 may switch off the pro-survival mode for the proper elimination of irreversibly damaged cells. In some cases, as a result of p53 activity, the homeostasis and integrity of the tissue as a whole is preserved. Interestingly, p73 (a p53 relative) was shown to enhance the PPP by activating the expression of G6PDH under conditions where p73 showed tumour-promoting activities (Du *et al.,* 2013). While illustrating the complexity of function of the p53 family of proteins, these studies support the general notion that flux through the PPP supports cancer cell growth.

#### (b) ATM kinase

PPP activity can be also stimulated by the kinase ataxia telangiectasia mutated (ATM). ATM is a serine/threonine kinase which is activated by DNA double-strand breaks and phosphorylates enzymes which are required for DNA checkpoint control and cell cycle arrest (Furgason & Bahassi, 2013). It phosphorylates the heat shock protein 27 (HSP27) which forms a complex with the first enzyme of the oxidative branch of the PPP: G6PDH. This interaction activates G6PDH and increases its activity supporting elevated PPP flux (Cosentino *et al.,* 2011) (Fig. 6). Therefore, by connecting genome stability and cell cycle control to PPP activation and metabolic adaptation, ATM represents another crucial hub for cellular homoeostasis during tumourigenesis (Cosentino *et al.,* 2011; Krüger & Ralser, 2011; Ditch & Paull, 2012).

#### (c) K-ras

The proto-oncogene *K-ras* is found activated in a number of human cancers, in particular adenocarcinomas of the pancreas, lung and colon (Downward, 2003). The observation that *K-ras*-transfected murine fibroblasts display enhanced resistance against oxidative stress *via* NADPH-mediated glutathione recycling first pointed towards a potential importance of the PPP for *K-ras*-induced transformation (Recktenwald *et al.,* 2008). Subsequently, it was found that oxidative stress is induced upon matrix detachment of cells and that resistance against detachment-induced cell death (anoikis) largely depends on antioxidant capacity (Schafer *et al.,* 2009). Intriguingly, anoikis resistance, which represents a central hallmark of malignant cells and a fundamental prerequisite for metastatic dissemination, in *K-ras*-driven human colon and mammary cancer cells, depends on functional integrity of the PPP (Weinberg *et al.,* 2010). While these results point towards a functional importance of PPP-mediated antioxidant capacity for *K-ras*-driven tumourigenesis, ROS generation has shown to be essential for the full oncogenic potential of *K-ras* (Weinberg *et al.,* 2010). These apparently contradictory results clearly need further experimental clarification before a clear-cut picture of the interplay between *K-ras* and the PPP during malignant transformation can be proposed (Fig. 6).

#### (d) HIF-1

The hypoxia-inducible transcription factor HIF-1 is found overexpressed in the majority of human cancers and regulates pivotal pro-tumourigenic features such as angiogenesis, glucose uptake and glycolysis as well as resistance towards apoptosis and anoikis (Rohwer *et al.,* 2013). Research on the role of HIF-1 for glucose metabolism was long dominated by HIF-1’s robust effect on glucose transport and glycolysis while experimental data supporting a role for HIF-1 in the control of cancer-associated PPP activity is intriguingly meagre. Analyses of the importance of oxidative stress during the pathogenesis of Alzheimer’s disease first reported a functional role of HIF-1 for enhanced PPP activity (Soucek *et al.,* 2003) (Fig. 6). Later, experimental evidence supported a critical role of HIF-1-mediated PPP activation in cellular antioxidant capacity of neuroblastoma cells (Guo *et al.,* 2009). The most compelling experimental evidence in this regard was published by Craig Thompson’s group: analysing chronic myeloid leukemia (CML) cells, they reported robust activation of HIF-1 in cells exhibiting resistance towards the tyrosine-kinase-inhibiting therapeutic imatinib (Zhao *et al.,* 2010). This HIF-1 activation was associated with reduced flux through the oxidative branch of the PPP while the glycolytic rate was significantly enhanced. On the other hand, the non-oxidative PPP branch was found activated in a TKL-dependent manner in cells with stabilized HIF-1, thereby supplying ribose synthesis essential for cellular proliferation (Zhao *et al.,* 2010). Chemical inhibition of TKL resulted in enhanced imatinib sensitivity *in vitro* and *in vivo* against CML, pointing towards a functional role of HIF-1-driven non-oxidative PPP in mediating resistance against targeted therapies. These results are especially intriguing as imatinib represents the only targeted therapeutic that was able to result in undisputed and long-lasting clinical benefit of patients with cancer. This work supports the notion of HIF-1 as a pivotal mediator of therapy failure and points towards HIF-1-dependent control of cellular metabolism as an important molecular mechanism (Rohwer & Cramer, 2011).

#### (e) PI3K-Akt/mTORC1

Aberrant activation of the signalling cascade comprising phosphatidyl inositol kinase (PI3K), protein kinase B (PKB/Akt) and the mammalian target of rapamycin complex 1 (mTORC1) is commonly observed in the majority of human cancers (Laplante & Sabatini, 2012). It became evident in recent years that mTORC1 exerts pro-tumourigenic activity not only *via* its well-established roles in protein synthesis and autophagy, but also *via* elaborate control over cellular metabolism. A genomic approach unravelled that mTORC1 induces a variety of genes that encode for specific metabolic pathways, e.g. glycolysis, lipid and sterol biosynthesis as well as both branches of the PPP (Düvel *et al.,* 2010). Despite these encouraging results, the functional importance of the PPP for mTORC1-driven cancers as well as the molecular nature of mTORC1 activation of PPP genes remains elusive. The antioxidant capacity needed to promote survival of tumour cells after detachment from the extracellular matrix depends on PI3K-Akt-induced activation of the oxidative PPP (Schafer *et al.,* 2009) (Fig. 6).

#### (f) Alternative pathways of PPP activation in cancer

The notion that increased PPP activity is beneficial for cancer cells is also supported by other studies that propose alternative mechanisms of PPP activation in cancer cells. For example, phosphofructokinase 1 (PFK1) is inhibited in cancer cells through glycosylation, drives PPP flux and supports cancer cell growth (Yi *et al.,* 2012). *Vice versa*, the depletion of 6-phosphofructo-2-kinase/fructose 2,6-bisphosphatase 4 (PFKFB4) inhibits cancer cell growth by lowering flux through the PPP (Ros *et al.,* 2012). Moreover, a study addressing the plant stilbenoid resveratrol indicates that its suppressive function on human colon cancer cell proliferation is attributable to PPP targeting and talin-focal adhesion kinase (talin-FAK) signalling pathways as well (Vanamala *et al.,* 2011).

### (5) Conclusions about the role of the PPP in cancer metabolism

While the prognosis of certain types of cancers (e.g. breast and colon cancer) has improved in recent years, we are still eagerly awaiting successful clinical translation of the billions of funding and an uncountable number of working hours that have been invested in cancer research in the last 40+ years. Otis W. Brawley, chief medical officer of the American Cancer Society, once said ‘One cancer cell is smarter than 100 brilliant cancer scientists’. We still need to unravel the basic principles that enable malignant transformation, unchecked proliferation, systemic spread and therapy resistance. There is good reason to believe that understanding cancer metabolism might provide an important contribution to these attempts. The study of the PPP could be central, as the pathway is at the crossroads of both oncogenic signalling and biosynthetic pathways. In this respect, first results are promising: in a recent study, PPP activity was predictive for the efficacy of cancer therapeutics (Folger *et al.,* 2011).

## IX. THE ROLE OF THE PPP IN BRAIN ENERGY METABOLISM

The brain energy demands to maintain its physiological signalling activities are extremely high. Although it represents only 2% of the total body mass, the adult human brain is believed to consume about 20% of oxygen respired at rest (Silver & Erecińska, 1998; Bélanger, Allaman & Magistretti, 2011). A developing brain might have even greater requirements, as estimates suggest that an infant’s brain can utilize more than 40% of basal metabolic rate (Goyal *et al.,* 2014). These large amounts of energy are needed for the maintenance and restoration of ionic gradients and for synaptic transmission (Attwell & Laughlin, 2001). The majority of ATP is generated through OXPHOS, therefore implying the strict reliance of neuronal activity on mitochondria functionality and oxygen supply (Ames, 2000; Erecinska, Cherian & Silver, 2004). Accordingly, mitochondrial impairment has a great impact on neuronal function and survival (Nicholls & Budd, 2000; Kann & Kovács, 2007), and it is considered a key pathogenic player in several neurodegenerative and neurodevelopmental disorders (Fiskum, Murphy & Beal, 1999; Beal, 2005).

**Fig. 7:**
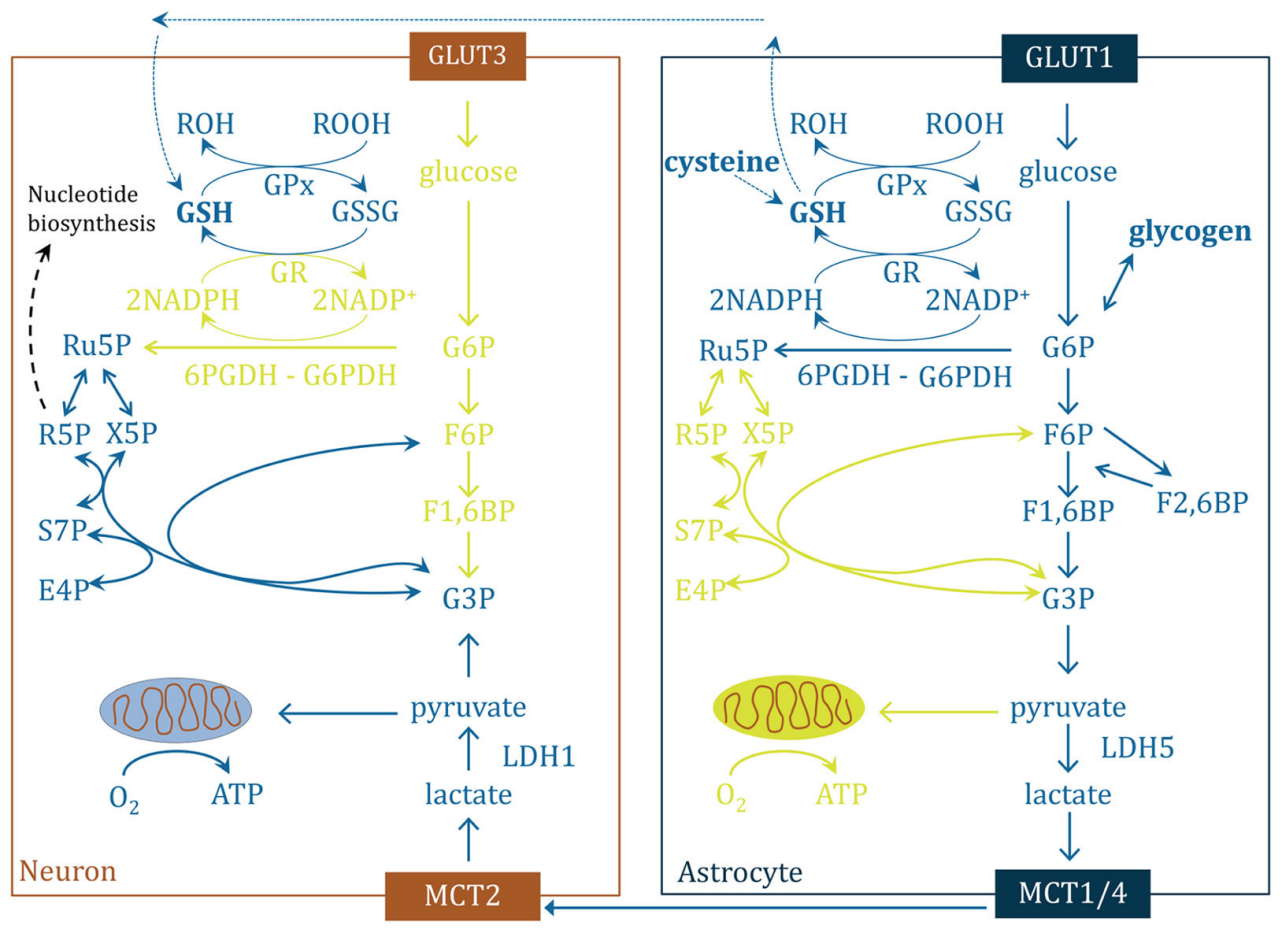
The pentose phosphate pathway (PPP) in neuronal energy metabolism. Schematic representation of glucose metabolism in neurons (left) and astrocytes (right). Metabolic regulators differentially expressed between neurons and astrocytes are highlighted. Summarized is evidence that astrocytes ferment glucose to lactate which is secreted aside GSH into the intracellular space. Neurons then uptake lactate and GSH; lactate is then converted to pyruvate and enters the tricarboxylic acid cycle to generate ATP over the respiratory chain. Abbreviations: as in Table 1; GSH, glutathione; GSSG, glutathione disulfide; GLUT, glucose transporters; GR, glutathione reductase; GPx, glutathione peroxidase; LDH, lactate dehydrogenase; MCT, monocarboxylate transporter.

Glucose represents the normal obligatory energy substrate of the brain, and 25% of its daily intake is assumed to be dedicated to cerebral functions (Cremer, 1982; Bélanger *et al.,* 2011). A steady glucose supply is necessary since the central nervous system (CNS) is able to store only a limited amount of glycogen within astrocytes (Brown & Ransom, 2007), glial cells outnumbering neurons in the human brain. Interestingly, quantitative measurements of whole-brain metabolism showed that about 10% of consumed glucose is in excess of oxygen utilization (Fox *et al.,* 1988). Therefore, glycolysis-based metabolism appears of fundamental importance for the energetic needs of active neuronal tissue. Studies of primary cultures of glia and neurons helped to demonstrate the physiological metabolic compartmentalization of the CNS. In particular, astrocytes are mainly glycolytic and convert glucose into lactate (Itoh *et al.,* 2003). This lactate can then be transferred to neurons *via* the so-called ‘astrocyte–neuron lactate shuttle’ and eventually employed by the neurons for OXPHOS-based ATP generation (Pellerin & Magistretti, 1994; Kasischke *et al.,* 2004). This model is supported by a cell-type-specific expression pattern of regulatory members of carbon metabolism (Lovatt *et al.,* 2007). These include glucose transporters (GLUT1 in astrocytes and GLUT3 in neurons), lactate dehydrogenase (LDH1 in astrocytes favouring lactate generation and LDH5 in neurons supporting pyruvate formation from lactate), and lactate transporter (monocarboxylate transporter MCT1/4 in astrocytes promoting lactate release and MCT2 in neurons promoting lactate uptake) (Fig. 7) (Bittar *et al.,* 1996; Ames, 2000; Bélanger *et al.,* 2011).

An interesting consequence of the metabolic coupling between astrocytes and neurons is the peculiar neuronal dependence on PPP activity. Indeed, lactate utilization as an oxidative substrate for energy production in neurons may represent a mechanism for circumventing glycolysis and thus sparing neuronal glucose for the PPP (Bolaños, Almeida & Moncada, 2010). In particular, a fundamental regulatory role has been proposed for the enzyme 6-phosphofructose-2-kinase/fructose-2,6-biphosphatase-3 (PFKFB3), which generates fructose 2,6-bisphosphate (F2,6BP), a potent activator of the rate-limiting glycolytic enzyme phosphofructokinase-1 (PFK1) (Hue & Rider, 1987). Due to constant proteasomal degradation, PFKFB3 is absent in neurons and cannot be activated upon inhibition of mitochondrial respiration (Almeida *et al.,* 2001; Herrero-Mendez *et al.,* 2009). On the contrary, PFKFB3 is expressed in astrocytes was upregulated upon mitochondrial impairment in order to increase the glycolytic rate (Herrero-Mendez *et al.,* 2009). Therefore, the high neuronal sensitivity to mitochondrial dysfunction may be due to their inability to sustain elevated glycolysis because of their dependence on PPP-based utilization of glucose. A similar mechanism may also be present in cancer cells, where PFKFB3 has been reported to display reduced methylation and enhanced degradation in the proteasome, resulting in the shunt of glucose away from glycolysis and towards the PPP (Yamamoto *et al.,* 2014).

The main reason underlying neuronal dependence on glucose metabolism *via* the PPP may be the maintenance of redox homeostasis (Fernandez-Fernandez, Almeida & Bolaños, 2012). Indeed, to counteract the increase in ROS, common by-products of OXPHOS, neuronal cells would need an antioxidant defence mechanism constantly in place. To this end, the production of NADPH within the oxidative branch of the PPP is critical, as it represents the main electron donor for the generation of reduced glutathione (GSH) through the enzyme glutathione reductase (GR). GSH is in turn employed as electron donor for the reduction of detrimental peroxides (ROOH) by glutathione peroxidase (GPx) (Dringen, 2000). Interestingly, although this process may be essential in neurons, it has been shown that astrocytes are better equipped to stimulate the PPP, and the consequent NADPH generation, in response to oxidative stress (Ben-Yoseph, Boxer & Ross, 1996*a*; García-Nogales, Almeida & Bolaños, 2003). Neurons are also less capable than astrocytes in utilizing extracellular cysteine, used as precursor of GSH, and thus rely on the uptake of GSH that has been produced and released by the astrocytes (Dringen, 2000). These data emphasize the susceptibility of neuronal cells to redox imbalance and their crucial necessity for PPP-based glucose metabolism (Ben-Yoseph, Boxer & Ross, 1996*b*). In accordance, brain PPP activity has been found induced upon experimental brain injury in mice and after traumatic brain injury in humans (Bartnik *et al.,* 2005; Dusick *et al.,* 2007). Furthermore, malfunction of the PPP is associated with the appearance of neurological symptoms (Herken, Lange & Kolbe, 1969).

Recent findings suggest a second reason behind the importance of the PPP in brain metabolism. A meta-analysis of glucose and oxygen consumption throughout the human lifespan and among different brain regions suggests that non-oxidative glucose utilization may be important during development to support synaptic remodelling (Vaishnavi *et al.,* 2010; Goyal *et al.,* 2014). This may imply that the nucleotide biosynthesis derived by PPP activity might be crucial in neurons for synaptic plasticity (Magistretti, 2014). Indeed, the PPP/glycolysis ratio has been found to be higher in neonatal brain compared to adult brain (Baquer *et al.,* 1977; Morken *et al.,* 2014).

The current model of neuron/astrocyte bioenergetics has been questioned by some groups (Dienel, 2012), as it has been shown that during network activation neurons may be as capable as astrocytes at employing glucose as an energy substrate (Ivanov *et al.,* 2014). Accordingly, glycolysis-generated ATP appears of fundamental importance for vesicle motility (Zala *et al.,* 2013). Therefore, as mitochondria may be unevenly distributed in the neuronal cells, the glycolytic machinery may provide the constant energy needed for fast axonal transport (Zala *et al.,* 2013).

Overall, our understanding of human brain energy metabolism is still limited. Perhaps, recent advances in stem cells and neuronal differentiation (Jakel, Schneider & Svendsen, 2004; Sandoe & Eggan, 2013) might be helpful in providing human CNS cells for the study of neuroenergetics at the cellular and molecular level. This might potentially clarify the role of the PPP in the CNS and the interplay between different human brain cell types in the basal state and under conditions stimulating remodelling of energy flux.

## X. CONCLUSIONS

The PPP is a central component of metabolism in the majority of single- and multicellular organisms. Despite the pathway is central and evolutionary ancient, it possesses a high level of flexibility, which renders it an attractive target for biotechnology and medicine. In summary

1. The main biochemical function of the PPP is the biosynthesis of nucleic-acid and amino-acid sugar phosphate precursors.
2. This function of the PPP is bound to the provision of biochemical reducing equivalents in form of NADPH, which renders the PPP an important player in maintaining redox homeostasis.
3. The PPP is highly flexible, dynamic, and is adapting to varying nutrient supply and stress conditions. This coordinates these functions and is required meet cellular metabolic demands in the constantly changing environment.
4. The PPP is important for biotechnology, as its flexibility can be exploited to tune NADPH production, and for medical research, as the PPP activity is altered by bacterial and eukaryotic parasites during the infection process, when stem cells differentiate, when cancer cells maintain redox homeostasis, and in neurons to sustain energy metabolism.
5. Unveiling the complex regulation of the PPP, which despite 80 years of detailed basic and medical research is still not fully understood, appears hence essential for addressing metabolic adaptation and its consequences on cellular and organismic physiology.

## Figures and Tables

**Table 1:** Enzymes of the cytosolic pentose phosphate pathway. PPP enzymes, enzyme commission (EC) number and the catalysed reaction

	Enzyme	Abbreviation	EC number	Reaction	References
**PPP enzymes**	Glucose 6-phosphate dehydrogenase	G6PDH	EC1.1.1.49	Glucose 6-phosphate + NADP^+^ ↔ 6-phospho-glucono-1,5-lactone + NADPH + H^+^	Warburg & Christian (1936) and Glaser & Brown (1955)
	6-Phosphogluconolactonase	6PGL	EC 3.1.1.31	6-Phosphoglucono-1,5-lactone + H_2_O → 6-phosphogluconate	Kawada *et al.* (1962) and Miclet *et al.* (2001))
	6-Phosphogluconate dehydrogenase	6PGDH	EC 1.1.1.44	6-Phosphogluconate + NADP^+^ → ribulose 5-phosphate + CO_2_ + NADPH + H^+^	Dickens & Glock (1951)
	Ribose 5-phosphate isomerase	RPI	EC 5.3.1.6	Ribulose 5-phosphate ↔ ribose 5-phosphate	Horecker, Smyrniotis & Seegmiller (1951)
	Ribulose 5-phosphate epimerase	RPE	EC 5.1.3.1	Ribulose 5-phosphate ↔ xylulose 5-phosphate	Dickens & Williamson (1956), Horecker & Hurwitz (1956) and Ashwell & Hickman (1957)
	Transketolase	TKL	EC 2.2.1.1	Sedoheptulose 7-phosphate + glyceraldehyde 3-phosphate ↔ ribose 5-phosphate + xylulose 5-phosphate	De La Haba, Leder & Racker (1955) and Horecker, Hurwitz & Smyrniotis (1956)
	Transaldolase	TAL	EC 2.2.1.2	Sedoheptulose 7-phosphate + glyceraldehyde 3-phosphate ↔ erythrose 4-phosphate + fructose 6-phosphate	Horecker & Smyrniotis (1955)
	Sedoheptulokinase	SHPK	EC 2.7.1.14	Sedoheptulose + ATP → sedoheptulose 7-phosphate + ADP	Ebata *et al.* (1955)) and Wamelink *et al.* (2008*b*)
	Sedoheptulose 1,7-bisphosphatase	SH17BPase	EC 3.1.3.37	Sedoheptulose 1,7-bisphosphate + H_2_O → sedoheptulose 7-phosphate + phosphate	Racker (1962) and Clasquin *et al.* (2011)
	Sedoheptulose 7-phosphate isomerase	SHI	EC 5.3.1.28	Sedoheptulose 7-phosphate ↔ glycero-manno-heptose 7-phosphate	Kneidinger *et al.* (2001) and Taylor *et al.* (2008)
**Glycolytic enzymes with PPP substrates (selection)**	Glucose phosphate isomerase	GPI	EC 5.3.1.9	Glucose 6-phosphate ↔ fructose 6-phosphate	Ramasarma & Giri (1956)
	Triosephosphate isomerase	TPI	EC 5.3.1.1	Glyceraldehyde 3-phosphate ↔ dihydroxy acetonephosphate (DHAP)	Meyerhof & Beck (1944)
	Glyceraldehyde 3-phosphate dehydrogenase	GAPDH	EC 1.2.1.12	Glyceraldehyde 3-phosphate + phosphate + NAD^+^ ↔ 1,3-bisphosphoglycerate + NADH + H+	Warburg & Cristian (1939)
